# Transdiagnostic mapping of common and specific regional homogeneity alterations across affective and psychotic disorders

**DOI:** 10.1038/s41386-026-02469-0

**Published:** 2026-06-25

**Authors:** Jialin Xiang, Junni Ran, Pengyu Zhu, Yuxi Wang, Xiumin Deng, Bo Yang, Lisha Zhang, Xiong Chen, Jiayi Guo, Jiaying Liu, Kun Qin, Wen Chen

**Affiliations:** 1 Department of Radiology, Taihe Hospital, Hubei University of Medicine, Shiyan, Hubei China; 2 Mental Health Center, Taihe Hospital, Hubei University of Medicine, Shiyan, Hubei China; 3 Huaxi MR Research Center (HMRRC), Department of Radiology, West China Hospital of Sichuan University, Chengdu, Sichuan China; 4 The National Clinical Research Center for Mental Disorders & Beijing Key Laboratory of Mental Disorders, Beijing Anding Hospital, Capital Medical University, Beijing, China

**Keywords:** Depression, Bipolar disorder, Schizophrenia

## Abstract

Major depressive disorder (MDD), bipolar disorder (BD) and schizophrenia (SZ) are generally categorized as distinct diagnoses, but shared clinical, biological and genetic features. Although rich neuroimaging evidence has revealed brain functional alterations in each of these disorders, the transdiagnostic patterns remain poorly understood. A comprehensive literature search was conducted up to March 2025 for resting-state functional MRI studies reporting regional homogeneity (ReHo) abnormalities in MDD, BD or SZ patients, as compared to healthy controls (HC). Voxel-wise meta-analyses of ReHo abnormalities were conducted separately for each disorder using the Seed-based d Mapping toolbox, followed by conjunction and contrast analyses to further identify common and disorder-specific patterns. Our meta-analysis included a total of 90 studies, encompassing 3764 patients (MDD: *n* = 1703; BD: *n* = 872; SZ: *n* = 1189) and 3590 HC. A transdiagnostic pattern of common ReHo increase was identified in the right inferior frontal gyrus, while disorder-specific ReHo alterations were distributed across different large-scale brain networks. Specifically, MDD-specific abnormalities were primarily involved the default mode and subcortical networks; BD-specific abnormalities were mostly identified within the default mode network; SZ patients exhibited more widespread dysfunction spanning across the frontoparietal, and somatomotor networks. These transdiagnostic findings advance the current understanding of neurofunctional mechanisms underlying overlapping and different multi-dimensional features across affective and psychotic disorders, offering novel insights into precision diagnostics and therapeutic targets for individualized psychiatry in the future.

## Introduction

Major depressive disorder (MDD), bipolar disorder (BD), and schizophrenia (SZ) are among the most prevalent and disabling psychiatric disorders, imposing a substantial social and economic burden worldwide [1, 2]. Although these affective and psychotic disorders have typically been classified as distinct diagnostic entities, they exhibit considerable overlap in clinical manifestations and treatment options [3]. In addition, substantial studies have further revealed convergence across these disorders at genetic, cellular, and molecular levels [4–6]. These findings challenge the traditional symptom-based diagnostic framework, fuelling debate over whether they represent distinct categories or exist along a continuum. Therefore, advancing the current understanding of transdiagnostic neurobiological substrates across these disorders is useful, which can accelerate the development of precise diagnostic markers and therapeutic targets.

Resting-state functional magnetic resonance imaging (rs-fMRI) has emerged as a non-invasive tool that plays a crucial role in elucidating the neural mechanisms underlying psychiatric disorders. Among multiple rs-fMRI metrics, regional homogeneity (ReHo), which assesses brain local activity synchronization, has been recognized as a robust neurofunctional measure owing to its high sensitivity and test-retest reliability [7–9]. Notably, a recent large-scale study reported that ReHo showed stronger case-control effects than cortical thickness and was strongly associated with rCBF deficits in MDD, supporting its potential as a functional biomarker candidate [10]. In addition, substantial neuroimaging studies have frequently reported ReHo abnormalities in patients with affective and psychotic disorders [11–13]. For example, MDD patients typically exhibited abnormal ReHo in prefrontal and occipital cortex [14, 15]. Substantial previous studies reported altered frontal, striatal, insular and temporal ReHo in BD [16]. Regarding SZ, studies have revealed aberrant ReHo in the precentral, occipital, dorsolateral prefrontal and insular cortices [17, 18].

Although previous studies have demonstrated that ReHo can serve as a potential biomarker for affective and psychotic disorders, the transdiagnostic insights from these findings remain elusive. Notably, a recent study has attempted to directly compare MDD and BD, finding that both groups showed decreased ReHo in parietal regions, whereas BD exhibited increased ReHo in occipital areas while MDD showed reductions in the putamen and angular gyrus [19]. Another study compared MDD and SZ, which revealed abnormalities in frontal and temporal regions, such as the superior frontal and superior temporal gyri, that appeared more specific to SZ [20]. A further study directly contrasted BD and SZ, which revealed widespread abnormalities in both disorders, with BD primarily involving frontal and temporal regions and SZ additionally engaging the cerebellum [21]. However, to date, no study has provided a systematic transdiagnostic comparison of ReHo abnormalities across MDD, BD, and SZ. Moreover, most findings from above single-site studies have been challenged by reproducibility issues with considerable inconsistency. Such heterogeneity may arise from intrinsic biological differences among patient populations, limited sample sizes, and variability in analytical methods. In this context, meta-analytic approaches can provide a powerful framework to address above limitations, enabling the synthesis of evidence accumulated across studies to derive potentially more reliable and generalizable conclusions regarding transdiagnostic patterns of ReHo abnormalities across disorders.

To address these limitations, the present study employed coordinate-based meta-analysis to integrate ReHo studies in MDD, BD, and SZ, with the aim of identifying transdiagnostic commonalities and disorder-specific features of local functional coherence alterations across these three disorders. Specifically, we conducted three independent meta-analyses for each disorder, followed by conjunction and contrast analyses to delineate shared and disorder-specific ReHo abnormalities across disorders. Our findings are expected to provide novel evidence for neural mechanisms underlying affective and psychotic disorders, thereby advancing the understanding of transdiagnostic neurobiological substrates.

## Materials and methods

### Literature search

A comprehensive search was conducted in the PubMed, Web of Science, and Embase databases for studies published between January 2004 and March 2025. The search terms included: “fMRI” OR “functional magnetic resonance imaging” OR “regional homogeneity” OR “ReHo”, in combination with the following categories of psychiatric disorders: (1) “schizophrenia” OR “psychotic” OR “psychosis”; (2) “bipolar” OR “manic” OR “mania”; and (3) “depress*” OR “MDD” OR “unipolar”. Studies were included if they (1) investigated ReHo abnormalities in MDD, BD or SZ patients relative to matched healthy controls (HC); (2) conducted whole-brain voxel-wise comparisons; and (3) provided peak coordinates in Montreal Neurological Institute (MNI) or Talairach space. Exclusion criteria included: (1) studies for which peak coordinates were unavailable and could not be obtained by contacting the authors; (2) studies reporting results only within predefined regions of interests or limited brain coverage; (3) preprints not peer-reviewed; and (4) non-empirical studies (e.g., reviews, meta-analyses, conference abstracts). For interventional studies with longitudinal follow-up, only baseline data were included to minimize treatment-related bias. If multiple studies had overlapping samples, the one with the largest sample size was selected for data extraction. In addition to original articles, reference lists of relevant reviews and meta-analyses were screened manually to ensure completeness. Our meta-analysis conformed to the Preferred Reporting Items for Systematic Reviews and Meta-Analysis (PRISMA) guidelines and was registered retrospectively on the Open Science Framework (OSF; 10.17605/OSF.IO/SWM69).

### Quality assessment and data extraction

Study quality was independently assessed using a 12-item checklist adapted from previous neuroimaging meta-analyses [22]. This checklist provides an integrative score considering quality of demographic and clinical characteristics, imaging methodology and results reporting (Supplementary Materials). Each item was scored as 1 (fully met), 0.5 (partially met), or 0 (not met). The quality of each study was independently assessed by two different authors (JR and XD). Discrepancies in ratings were discussed with a senior author (KQ) to reach a consensus. From each included study, two authors (JX and KQ) extracted the peak coordinates of clusters showing significant between-group ReHo differences, along with the t-statistics. Other available study information, such as sample size, mean age and sex ratio, illness duration and age of onset was also extracted.

### Coordinate-based meta-analysis of neuroimaging studies

Our meta-analyses were conducted using the anisotropic effect-size version of Signed Differential Mapping (AES-SDM, version 5.15) toolbox [23]. This method has been widely applied in prior neuroimaging meta-analyses, and its methodological details have been described elsewhere [24, 25]. In brief, AES-SDM uses peak coordinates and corresponding statistical values to reconstruct whole-brain maps of effect sizes and variances at the voxel level. By implementing a random-effects model, it allows for the integration of individual studies weighted by sample size and intra-study variance. The main analytical steps include data organization, preprocessing, voxel-wise meta-analysis, and statistical thresholding. We first conducted single-disorder meta-analyses separately to examine significant ReHo abnormalities in MDD, BD and SZ. Consistent with the previous studies [26–28], the threshold of meta-analysis was set as an uncorrected voxel-wise *p* < 0.005, SDM-Z > 1 and cluster size >10 voxels to get significant clusters with optimal balance between sensitivity and specificity [25]. Between-study heterogeneity and publication bias were evaluated for each single-disorder meta-analytic finding (Supplementary Materials). In addition, Jackknife sensitivity analyses were performed to assess the robustness of findings (Supplementary Materials).

### Analyses of shared and disorder-specific ReHo abnormalities

Shared (i.e., observed consistently across all three disorders) and disorder-specific (i.e., significantly stronger or weaker in one disorder compared to the other two) patterns of ReHo abnormalities were examined across MDD, BD and SZ. First, we obtained effect size maps of ReHo abnormalities for each disorder from the corresponding single-disorder meta-analyses. Subsequently, conjunction and contrast analyses were performed between these maps using the SDM built-in “multimodal” and “linear model” functions, respectively. Specifically, the “multimodal” function enabled conjunction analysis to robustly quantify shared neural patterns between two meta-analytic maps [29], while the “linear model” function compares effect sizes between two meta-analyses and generates contrast maps showing regions where abnormal patterns significantly differed [30].

For shared ReHo abnormalities, we adopted a stepwise approach [31]: (1) first-level conjunction which generated a meta-analytic map of shared ReHo alterations between MDD and BD; (2) second-level conjunction integrating the above MDD-BD map with the SZ map to derive the final conjunctive map of tri-disorder ReHo abnormalities. Given the inherently four-tailed nature of the conjunction test, a more stringent voxel-level threshold of *p* < 0.0025 was applied in accordance with SDM recommendations [29]. To ensure the robustness of our findings to the order of the stepwise conjunction, we repeated the analysis using alternative sequences [(MDD & SZ) & BD, and (BD & SZ) & MDD]. We found the final results remained largely consistent regardless of the conjunction sequence used (Fig. S1).

To identify disorder-specific patterns, we employed a two-stage analytical approach: (1) pairwise contrast analysis: differential ReHo abnormalities between each pair of disorders were computed to first obtain contrast maps e.g., a greater ReHo increase in MDD compared to BD, represented as “MDD > BD”; (2) conjunction analysis of contrast maps*:* To isolate disorder-specific effects, conjunction analyses were performed across contrast maps involving the same disorder, focusing only on those with consistent effect directions [e.g., (MDD > BD) ∩ (MDD > SZ), indicating a greater ReHo increase in MDD relative to both BD and SZ]. The same statistical thresholds as those used in the shared effects analysis were applied. To ensure the identified disorder-specific patterns represent real abnormalities relative to HC, rather than merely reflecting relative differences between one disorder and the other two, the derived clusters were further masked using the corresponding single-disorder meta-analytic abnormality maps.

### Large-scale network mapping

We characterized the network-level distribution of ReHo abnormalities by mapping the meta-analytic findings onto canonical large-scale functional networks, as defined by Yeo et al. [32], including default mode network (DMN), frontoparietal network (FPN), limbic network (LIM), ventral attention network (VAN), dorsal attention network (DAN), somatomotor network (SMN), and visual network (VIS). In addition to these seven cortical networks, we also included the subcortical network (SUB) to account for abnormalities in subcortical structures. The subcortical components contained bilateral nucleus accumbens, amygdala, hippocampus, thalamus, putamen, pallidum and caudate. The network distribution percentage was calculated as the ratio of significant voxels overlapping with each network to the total number of significant voxels [33]. Non-parametric permutation tests were conducted to evaluate the statistical significance of network distribution percentages. Specifically, for each significant effect, spatially constrained permutation was applied to generate random brain maps that preserved spatial autocorrelation [34]. The network distribution percentage was then recalculated for each permuted brain map. This procedure was iterated 1000 times to construct an empirical null distribution representing the scenario of no preferential network alignment. The significance of the observed network distribution percentage was then assessed by comparing it against this null distribution. The threshold for permutation tests across the 8 networks was corrected for multiple comparisons using the Bonferroni method (*p* < 0.05/8).

### Ancillary analyses

To explore whether demographic and clinical factors showed modulatory effects on ReHo abnormalities in each disorder, meta-regression analyses were performed on a few study-level variables including mean age, sex ratio, illness duration and age of onset. Other variables that were available in less than 10 studies were not considered for the reliability of our results. In line with previous meta-analyses [35–37], a more stringent threshold was applied (voxel-level *p* < 0.0005) to reduce the risk of type I errors [24, 25]. Reliability analyses were further conducted to assess the potential confounding effects of methodological factors, including magnetic field strength, smoothing kernel size, ReHo calculation approach and medication status. Specifically, we re-ran the primary analyses in each group while restricting the included studies to those using a 3 T scanner, a smoothing kernel of <8 mm, and the Kendall’s W method with 27 neighbouring voxels (3 × 3 × 3). Subgroup analyses focusing on non-medicated samples were conducted for MDD and SZ, where at least 10 studies were available. In addition, findings from pairwise inter-disorder comparisons were extracted from the main analysis to inform common and distinct ReHo abnormalities across diagnostic groups (i.e., MDD vs BD, BD vs SZ, MDD vs SZ).

## Results

### Included studies and sample characteristics

**Fig. 1:**
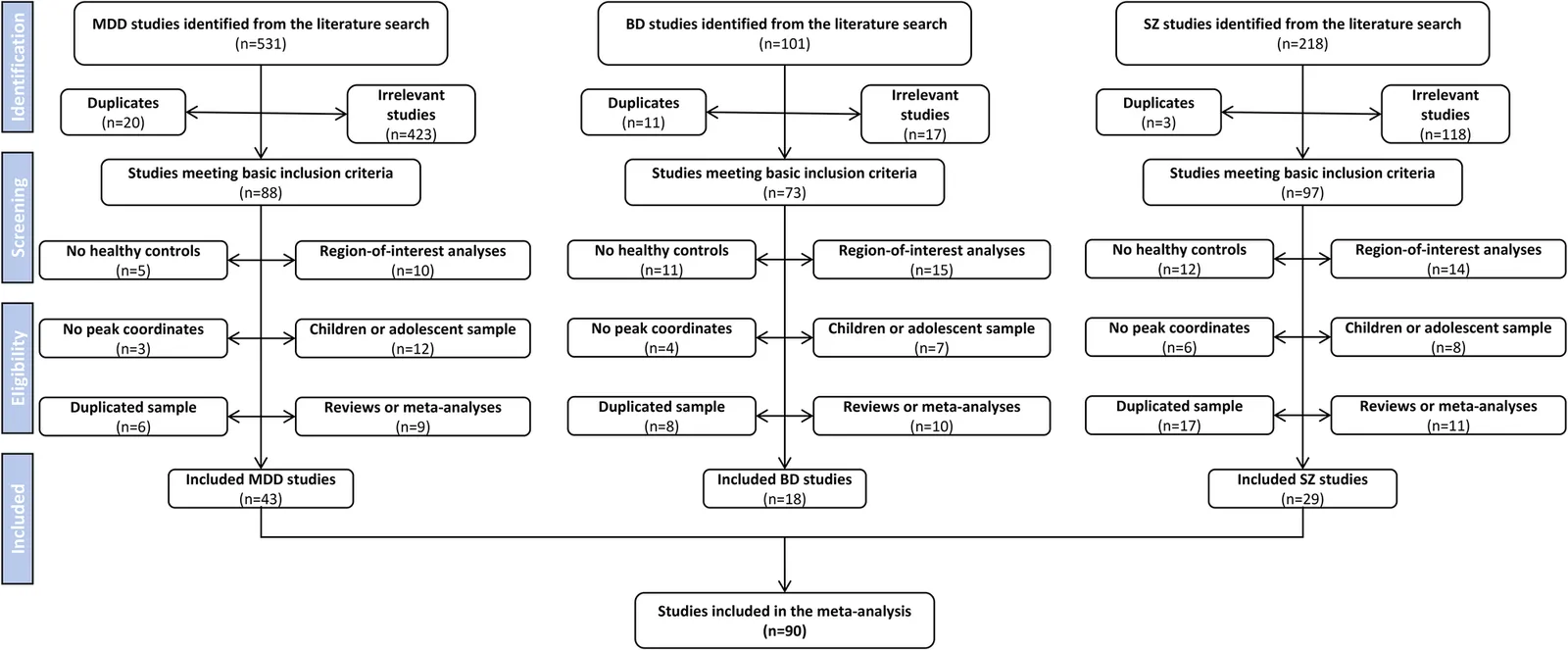
Study selection flowchart of the current meta-analysis.

A total of 43 studies on MDD (patients: *n* = 1703, mean age = 31.1, 59.8% females), 18 studies on BD (patients: *n* = 872, mean age = 30.2, 56.7% females), and 29 studies on SZ (patients: *n* = 1189, mean age = 25.9, 44.3% females) were finally included in the meta-analysis (Fig. 1 and Table 1). In all included studies, patients were matched to HC in terms of age and sex. The mean quality scores for studies on MDD, BD, and SZ were 11.65, 11.58, and 11.56, respectively, indicating generally high methodological quality. Detailed information on the included studies for each disorder is presented in Tables S1–S3.

**Table 1:** Demographic and clinical characteristics of all included MDD, BD and SZ studies.

Variable	MDD study		BD study		SZ study	
	Patients	HC	Patients	HC	Patients	HC
Sample size, N	1703	1685	872	834	1189	1071
Age, years	31.06 ± 11.24	31.19 ± 10.88	30.15 ± 13.68	27.66 ± 10.92	25.89 ± 9.63	27.36 ± 9.67
Sex, N female (%)	1019 (59.8%)	999 (59.3%)	494 (56.7%)	489 (58.6%)	527 (44.3%)	486 (45.4%)

*MDD* major depressive disorder, *BD* bipolar disorder, *SZ* schizophrenia, *HC* healthy controls.

### Single-disorder patterns of ReHo abnormalities

**Fig. 2:**
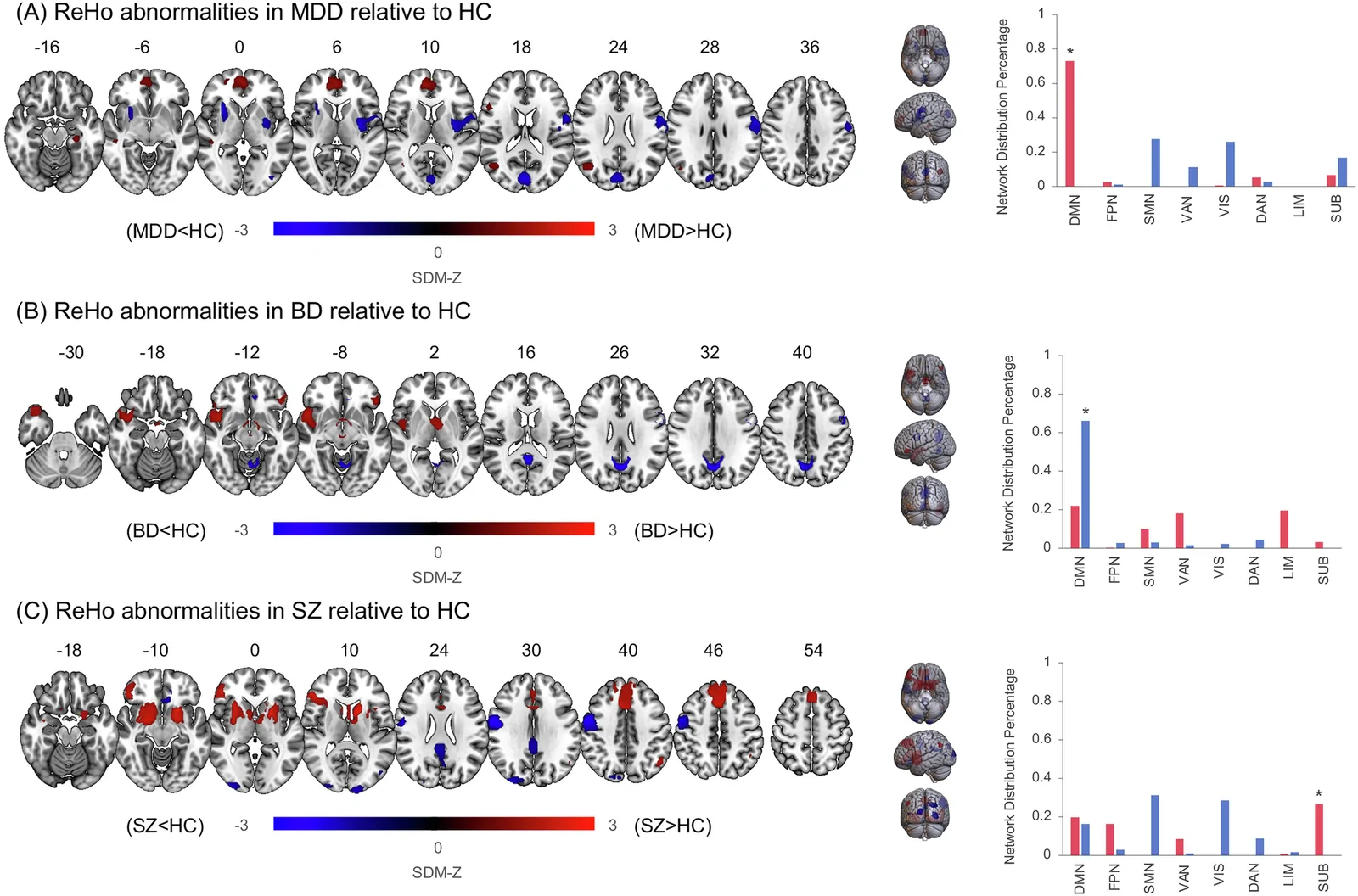
Single-disorder patterns of ReHo abnormalities and their corresponding network-level distributions. ReHo abnormalities and corresponding network distribution in MDD (**A**), BD (**B**) and SZ (**C**) patients compared to HC. Note: The network distribution percentage was calculated as the ratio of the number of voxels that the identified cluster overlapped with the network to the voxel size of the whole cluster. The colour bar indicates regional changes in ReHo: red denotes increases and blue denotes decreases. The same colour gradient scale is applied to both the brain slice views and the glass brain images. Numbers above slices indicate Z coordinates in MNI space. Asterisks (*) indicate statistically significant network distribution percentages. MDD major depressive disorder, BD bipolar disorder, SZ schizophrenia, HC healthy controls, ReHo regional homogeneity, FPN frontoparietal network, DAN dorsal attention network, DMN default mode network, LIM limbic network, SMN somatomotor network, VAN ventral attention network, VIS visual network, SUB subcortical network.

Compared with HC, MDD patients exhibited increased ReHo in the medial prefrontal cortex (mPFC), anterior cingulate cortex (ACC), inferior frontal gyrus (IFG) and temporal cortex, alongside decreased ReHo in the somatomotor areas, occipital cortex and striatum (Fig. 2A and Table S4). For BD patients, ReHo was increased in the insula, temporal pole and IFG, while decreased in the precuneus, cerebellum and precentral gyrus (Fig. 2B and Table S5). SZ patients, compared with HC, showed ReHo increases in the striatum, IFG, mPFC and angular gyrus. ReHo decreases in SZ were mainly observed in the somatomotor areas, posterior cingulate cortex (PCC) and occipital cortex (Fig. 2C and Table S6). Large-scale network mapping further identified significantly enriched networks within single-disorder patterns of ReHo abnormalities, including the DMN (73.1%, *p* < 0.001) in MDD, the DMN (66%, *p* < 0.001) in BD, and the SUB (26.6%, *p* < 0.001) in SZ.

### Common and disorder-specific ReHo abnormalities across MDD, BD and SZ

**Fig. 3:**
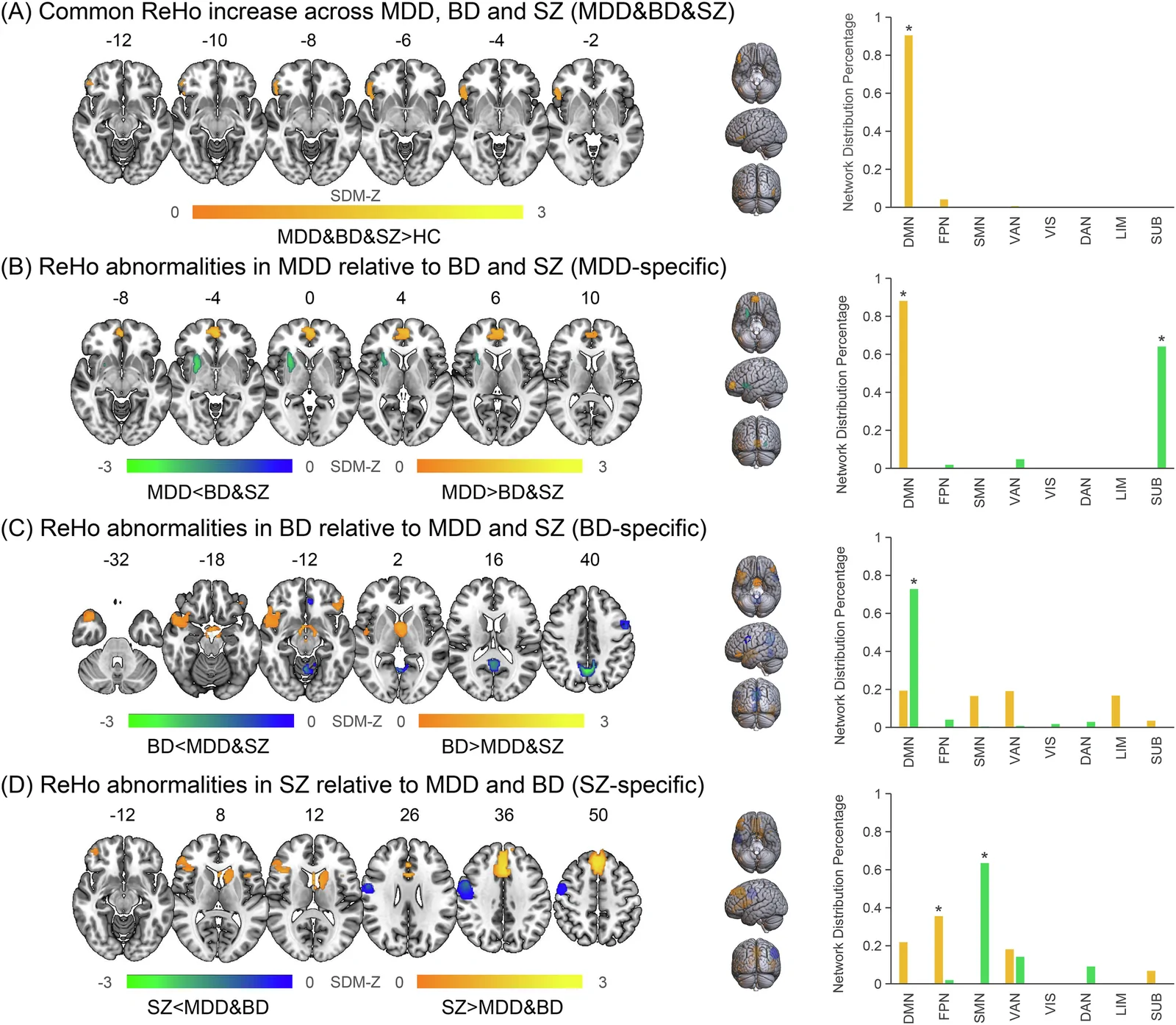
Common and disorder-specific patterns of ReHo abnormalities across MDD, BD and SZ. **A** Common ReHo increase across MDD, BD and SZ (MDD&BD&SZ); **B** ReHo abnormalities in MDD relative to BD and SZ (MDD-specific); **C** ReHo abnormalities in BD relative to MDD and SZ (BD-specific); **D** ReHo abnormalities in SZ relative to MDD and BD (SZ-specific). Note: The network distribution percentage was calculated as the ratio of the number of voxels that the identified cluster overlapped with the network to the voxel size of the whole cluster. The colour scheme denotes regional changes in ReHo: increases are represented in yellow, and decreases are shown in green. The same colour gradient scale is applied to both the brain slice views and the glass brain images. Numbers above slices indicate Z coordinates in MNI space. Asterisks (*) indicate statistically significant network distribution percentages. MDD major depressive disorder, BD bipolar disorder, SZ schizophrenia, ReHo regional homogeneity, FPN frontoparietal network, DAN dorsal attention network, DMN default mode network, LIM limbic network, SMN somatomotor  network, VAN ventral attention network, VIS visual network, SUB subcortical network.

Common patterns of ReHo increase across MDD, BD and SZ was identified in the rIFG with significant enrichment in the DMN (90.5%, *p* = 0.001; Fig. 3A and Table 2). There was no common ReHo decrease across the three disorders. For disorder-specific patterns, MDD patients showed greater ReHo increase in the ACC and orbitofrontal cortex (OFC), along with a weaker ReHo decrease in the putamen (Fig. 3B and Table 2). BD patients exhibited more pronounced ReHo increases in the temporal pole, insula and OFC, and stronger ReHo decrease in the precuneus, cerebellum and precentral gyrus (Fig. 3C and Table 2). SZ patients showed greater ReHo increase in the IFG, caudate and mPFC, and more marked ReHo decrease in the precentral and postcentral gyrus (Fig. 3D and Table 2). Large-scale network mapping further identified different networks significantly enriched for disorder-specific ReHo abnormalities: the DMN (88.1%, *p* < 0.001) and SUB (64.1%, *p* = 0.003) for MDD, the DMN (72.8%, *p* = 0.001) for BD, and the FPN (35.6%, *p* = 0.004) and SMN (63.5%, *p* < 0.001) for SZ.

**Table 2:** Common and specific patterns of ReHo abnormalities across MDD, BD and SZ.

Region	Breakdown	Voxels	
***Common ReHo increase(MDD&BD&SZ*** > ***HC)***			
Right inferior frontal gyrus	Right inferior frontal gyrus, orbital part Right inferior frontal gyrus, opercular part		168
			16
***MDD-Specific ReHo increase(MDD*** > ***BD&SZ)***			
Anterior Cingulate Cortex, pregenual part/Medial Orbitofrontal/Medial Superior Frontal Gyrus	Left anterior cingulate cortex, pregenual part		122
	Right medial orbital part of the superior frontal gyrus		83
	Right medial part of the superior frontal gyrus		70
	Right anterior cingulate cortex, pregenual part		59
	Left medial part of the superior frontal gyrus		41
	Left medial orbital part of the superior frontal gyrus		30
***MDD-Specific ReHo decrease(MDD*** < ***BD&SZ)***			
Right putamen	Right putamen		220
***BD-Specific ReHo increase(BD*** > ***MDD&SZ)***			
Right superior temporal pole /Right insula/Right orbitofrontal cortex	Right superior temporal pole		429
	Right insula		356
	Right superior temporal gyrus		48
	Right inferior frontal gyrus, orbital part		27
	Right posterior orbitofrontal cortex		25
	Right middle temporal pole		24
Left inferior frontal gyrus, orbital part	Left inferior frontal gyrus, orbital part		125
***BD-Specific ReHo decrease(BD*** < ***MDD&SZ)***			
Cerebellum/Left lingual gyrus	Cerebellar vermis (lobules IV–V)		106
	Left cerebellar hemisphere (lobules IV–V)		57
	Left lingual gyrus		26
Right precuneus	Left precuneus		367
	Right precuneus		320
	Left posterior cingulate gyrus		36
Left precentral gyrus	Left precentral gyrus		25
***SZ-Specific ReHo increase(SZ*** > ***MDD&BD)***			
Inferior Frontal Gyrus	Right inferior frontal gyrus, triangular part		375
	Right inferior frontal gyrus, orbital part		38
	Right middle frontal gyrus		38
Left caudate nucleus	Left caudate nucleus		281
Medial superior frontal gyrus/Middle cingulate gyrus/Supplementary motor area/Left anterior cingulate gyrus, supragenual part	Left medial superior frontal gyrus		668
	Right medial superior frontal gyrus		344
	Right middle cingulate gyrus		206
	Left middle cingulate gyrus		103
	Left supplementary motor area		92
	Left anterior cingulate gyrus, supragenual part		53
	Right supplementary motor area		32
***SZ-Specific ReHo decrease (SZ*** < ***MDD&BD)***			
Right precentral gyrus/Right postcentral gyrus	Right precentral gyrus		583
	Right postcentral gyrus		411

*ReHo* regional homogeneity, *MDD* major depressive disorder, *BD* bipolar disorder, *SZ* schizophrenia, *HC* healthy control.

### Meta-regression and reliability analyses

Meta-regression analysis revealed that in MDD patients, age was positively associated with ReHo alteration in the right middle temporal gyrus, and negatively associated with ReHo alterations in the bilateral occipital cortex and right postcentral gyrus. In addition, illness duration in MDD was negatively associated with ReHo alteration in the medial orbitofrontal cortex, while age of onset showed a negative association with ReHo alteration in the left middle occipital gyrus (Fig. S5*,* Table S7). In SZ, age and illness duration were negatively correlated with ReHo alterations in the occipital and parietal regions (Fig. S6*,* Table S7). These findings suggest that ReHo alterations are partially modulated by demographic variables, indicating a potential role of these factors in shaping functional brain abnormalities in psychiatric disorders. For the reliability analyses, patterns of ReHo abnormalities were substantially consistent when controlling for magnetic field strength, smoothing kernel size, and ReHo calculation approach (Tables S14–S16), suggesting these factors are unlikely to materially affect our main findings. Similarly, subgroup analyses restricted to non-medicated MDD and SZ samples showed largely consistent ReHo alteration patterns with the primary analyses, with core abnormalities remaining significant despite minor variations in cluster extent or peak statistics (Tables S17–S18). Common and distinct patterns of ReHo abnormalities for each pair of disorders (MDD vs. BD, BD vs. SZ, MDD vs. SZ) are presented in the Supplementary Results (Tables S8–S10; Figs. S2–S4).

## Discussion

Our study is the first meta-analysis to integrate evidence for ReHo abnormalities in MDD, BD, and SZ from a transdiagnostic perspective and to identify common and disorder-specific patterns of altered spontaneous brain activity across these disorders. Our results demonstrated that ReHo was consistently increased in the rIFG across the three disorders, while no common ReHo decreases were observed. Disorder-specific analyses revealed that MDD was characterized by greater ReHo in the ACC and OFC, as well as milder decreases in the putamen, relative to BD and SZ. In contrast, BD was characterized by more extensive ReHo increases across limbic and temporal regions, together with stronger decreases in the precuneus, cerebellum and precentral gyrus. SZ was characterized by greater ReHo in frontostriatal and medial prefrontal regions, along with reductions in the primary sensorimotor cortices. This pattern of shared and disorder-specific ReHo abnormalities may reflect overlapping but distinct pathophysiological processes across affective and psychotic disorders. Before interpreting the above findings, it is important to note that ReHo, computed using Kendall’s coefficient of concordance, indexes the local functional synchrony of resting-state BOLD time series among neighbouring voxels [7]. Accordingly, increased ReHo reflects greater local temporal coherence of intrinsic activity, whereas decreased ReHo indicates reduced local synchrony [38]. Importantly, as an indirect hemodynamic metric, ReHo captures alterations in local intrinsic coordination rather than overall activity levels, long-range connectivity, or specific neurobiological mechanisms [8].

The single-disorder meta-analyses revealed that ReHo abnormalities in affective and psychotic disorders were mainly distributed within the prefrontal, temporal, limbic/paralimbic, and subcortical systems, which are integral components of the cortico-striato-thalamo circuitry. This large-scale circuit plays a pivotal role in emotional regulation, cognitive control, and reward processing [39], and its dysfunction has been widely reported across major neuropsychiatric disorders [40–42]. These findings suggest that disrupted local synchronization within these regions may constitute a shared neurophysiological substrate underlying MDD, BD, and SZ. Notably, patients with SZ exhibited more extensive and pronounced ReHo abnormalities compared with those with MDD and BD. This observation is consistent with previous neuroimaging evidence showing greater effect sizes for both structural and functional abnormalities in SZ relative to affective disorders [43, 44]. We speculate that such neurophysiological distinctions may reflect the divergent neural mechanisms underlying distinct clinical phenotypes, particularly the more severe neurocognitive impairment and psychosocial dysfunction observed in SZ [45], which warrants further investigation in future studies.

At the transdiagnostic level, increased ReHo of the rIFG was consistently found in MDD, BD, and SZ and thus suggests that this region may be a universal neural vulnerability node for affective and psychotic psychopathology. The rIFG is important for impulse control, attentional regulation, and reward processing [46, 47], which are involved in core clinical symptoms and cognitive impairments frequently observed in MDD, BD, and SZ patients. Higher functional connectivity and ReHo in the right rIFG (segregating the triangularis) were previously demonstrated in patients with MDD [48], with positive correlations with symptom severity [49]. Furthermore, two large numbers of multicenter studies by the ENIGMA consortium revealed significant cortical thinning of the rIFG in SZ as well as BD patients [50, 51]. These observed structural and functional abnormality patterns were highly consistent with the critical role of the rIFG in cognitive control, particularly inhibitory control [52, 53]. The rIFG has recently been explored as a stimulation target using both invasive and noninvasive neuromodulation techniques, such as direct electrical stimulation (DES) and transcranial magnetic stimulation (TMS) [54, 55], with accumulating evidence suggesting that modulation of this region can enhance inhibitory control and executive functioning, particularly in ADHD [56]. Although further validation is warranted, in light of our findings, the rIFG may be considered an emerging target for transdiagnostic interventions across MDD, BD, and SZ, potentially facilitating the selective improvement of shared cognitive and inhibitory control deficits.

Compared with BD and SZ, MDD exhibited a more focal pattern of ReHo abnormalities, characterized by selective increases in the ACC and OFC and relatively mild decreases in the putamen. This focal enhancement within prefrontal-limbic territories, together with relative preservation of the striatum, suggests that the pathophysiology of MDD may primarily involve localized hyper-synchronization within emotion-regulatory circuits rather than widespread network disruption. This pattern is consistent with previous evidence indicating that the ACC and OFC play central roles in affective evaluation and cognitive control [57–59], supporting the notion that MDD is predominantly characterized by an imbalance in top-down emotional regulation rather than large-scale cortical disorganization [60].

Disorder-specific ReHo increases in BD were primarily located in the temporal pole, insula, and OFC, regions broadly belonging to the fronto–limbic system. Fronto–limbic dysfunction is considered a core neural circuitry underlying the pathophysiology of BD [61] and has also been reported in offspring of individuals with BD [62]. In contrast, ReHo decreases in BD showed significant enrichment in the DMN. The precuneus, a core hub of the DMN, exhibited reduced local synchrony, which may reflect instability in internally directed cognitive-affective processing in BD [63]. Additionally, decreased ReHo in the cerebellum and somatomotor cortex suggests that BD-related abnormalities may extend to motor-related systems, consistent with previous reports of disrupted cerebro–cerebellar connectivity and somatomotor network dysfunction in BD [64].

Compared with BD and MDD, SZ exhibited the most spatially extensive and hierarchically distributed pattern of local dysregulation, involving both higher-order associative and lower-level sensorimotor systems. This co-occurrence of frontal-striatal hyper-synchrony and sensorimotor hypo-synchrony suggests a breakdown in cortico-subcortical integration, aligning with neurobiological models emphasizing dysregulated dopaminergic signalling and impaired top-down control in psychosis [65, 66]. Notably, the striatal clusters showing increased ReHo in SZ exhibited substantial between-study heterogeneity; therefore, interpretations of fronto-striatal hypersynchrony should be made with appropriate caution. The abnormal enhancement of ReHo in frontostriatal circuits may underlie cognitive and motivational deficits, whereas reduced coherence in the SMN may contribute to the marked psychomotor retardation and disturbed motor coordination frequently observed in schizophrenia. Collectively, these findings indicate that schizophrenia is characterized by large-scale dysintegration across cortical and subcortical systems, distinguishing it from the more restricted and network-confined alterations seen in BD and MDD [67, 68].

Although this study provides integrative evidence of cross-diagnostic functional brain abnormalities, several limitations should be acknowledged when interpreting the findings. First, some of the included studies did not rigorously control for psychotropic medication use. Psychotropic medication remains a potential confound due to heterogeneous exposure and inconsistent control across studies. However, subgroup analyses in non-medicated MDD and SZ studies showed largely consistent spatial patterns with the primary results, suggesting that the main findings are not likely to be driven by medication effects. Nevertheless, residual confounding effects cannot be fully excluded, and a subgroup analysis was not feasible for BD due to limited data. Second, we employed a coordinate-based rather than image-based meta-analysis, which imposes limitations on the accuracy of reconstruction of effect size maps. In addition, given differences in the selection of included studies, the results of our single-disorder meta-analysis were not entirely consistent with some previous reports [16, 69], indicating that coordinate-based neuroimaging meta-analysis may be sensitive to variations in study inclusion. In light of the inherent heterogeneity of psychiatric disorders, establishing standardized and unified criteria for study inclusion and quality assessment would help improve the robustness of future neuroimaging meta-analysis. Third, all the original studies included in this meta-analysis were based on cross-sectional designs, restricting our ability to examine the longitudinal progression and causal relationships of brain functional alterations. Another limitation is that this meta-analysis was registered retrospectively rather than prospectively, which may limit the transparency of a priori analytic planning. Finally, the exploratory meta-regression findings suggest that demographic and clinical heterogeneity across studies may partly contribute to the inconsistent ReHo abnormalities reported in the literature. Heterogeneous or even opposing effects across samples may attenuate pooled effect sizes, thereby increasing the possibility of null or false-negative findings. However, due to insufficient reporting in the original studies regarding participants’ comorbid conditions, mood states, or diagnostic subtypes, we were unable to control for these heterogeneous variables or conduct subgroup analyses. Future research should emphasize multicenter longitudinal designs, standardized clinical data acquisition and individual-level data sharing to improve the stability and translational value of neuroimaging biomarkers in psychiatric disorders.

In summary, this transdiagnostic meta-analysis revealed both common and disorder-specific patterns of ReHo abnormalities across MDD, BD, and SZ. The rIFG exhibited consistently increased ReHo across all three disorders, suggesting that it may serve as a potential cross-diagnostic locus of abnormality, reflecting shared neural correlates underlying affective and psychotic psychopathology. At the disorder-specific level, MDD showed relatively localized abnormalities mainly confined to the prefrontal-limbic circuits. In BD, distinct nodes within the same large-scale network exhibited both increased and decreased ReHo, indicating intra-network dyscoordination of local synchrony. In contrast, SZ demonstrated the most spatially extensive abnormalities, encompassing both cortical and subcortical regions and extending into the sensorimotor network, reflecting a more widespread pattern of network dysregulation. Overall, these findings suggest that affective and psychotic disorders may share partially overlapping neural substrates while differing in the extent, hierarchy, and topography of network dysfunction. Future longitudinal and mechanistic studies are warranted to validate these findings and to evaluate their potential implications for diagnostic stratification and the development of circuit-based, cross-diagnostic intervention strategies.

## Supplementary information

Supplementary

## Data Availability

The datasets generated during and/or analyzed during the current study are available from the corresponding author on reasonable request.
