# Supplementary material for: Transdiagnostic mapping of common and specific regional homogeneity alterations across affective and psychotic disorders
Source: Neuropsychopharmacology. 2026 Jun 25;51(11):2093–101. doi: 10.1038/s41386-026-02469-0 (PMC13598073; doi:10.1038/s41386-026-02469-0)
Supplement: Supplementary file 1 [file 41386_2026_2469_MOESM1_ESM.docx]

**Online Supplementary Information**

**Transdiagnostic Mapping of Common and Specific Regional Homogeneity Alterations Across Affective and Psychotic Disorders**

**SUPPLEMENTARY METHODS**

I. Quality Assessment Checklist

II. Neuroimaging meta-analysis implemented by AES-SDM

III. Heterogeneity, Jackknife Sensitivity, Publication Bias

**SUPPLEMENTARY RESULTS**

I. Common and distinct ReHo abnormalities between MDD and BD

II. Common and distinct ReHo abnormalities between BD and SZ

III. Common and distinct ReHo abnormalities between MDD and SZ

IV. Heterogeneity, Jackknife Sensitivity, Publication Bias

**SUPPLEMENTARY TABLES**

**Table S1** Demographic and clinical characteristics of included MDD studies.

**Table S2** Demographic and clinical characteristics of included BD studies.

**Table S3** Demographic and clinical characteristics of included SZ studies.

**Table S4** Meta-analytic findings on ReHo abnormalities in MDD.

**Table S5** Meta-analytic findings on ReHo abnormalities in BD.

**Table S6** Meta-analytic findings on ReHo abnormalities in SZ.

**Table S7** Meta-regression findings.

**Table S8** Common and distinct patterns of ReHo abnormalities between MDD and BD.

**Table S9** Common and distinct patterns of ReHo abnormalities between BD and SZ.

**Table S10** Common and distinct patterns of ReHo abnormalities between MDD and SZ.

**Table S11** Jackknife sensitivity analysis for MDD meta-analysis.

**Table S12** Jackknife sensitivity analysis for BD meta-analysis.

**Table S13** Jackknife sensitivity analysis for SZ meta-analysis.

**Table S14** Reliability analyses of meta-analytic ReHo abnormalities in MDD.

**Table S15** Reliability analyses of meta-analytic ReHo abnormalities in BD.

**Table S16** Reliability analyses of meta-analytic ReHo abnormalities in SZ.

**Table S17** Subgroup analysis of ReHo abnormalities in non-medicated MDD patients.

**Table S18** Subgroup analysis of ReHo abnormalities in non-medicated SZ patients.

**SUPPLEMENTARY FIGURES**

**Figure S1** Shared ReHo results across different conjunction sequences.

**Figure S2** Common and distinct patterns of ReHo abnormalities between MDD and BD.

**Figure S3** Common and distinct patterns of ReHo abnormalities between BD and SZ.

**Figure S4** Common and distinct patterns of ReHo abnormalities between MDD and SZ.

**Figure S5** Meta-regression findings in MDD.

**Figure S6** Meta-regression findings in SZ.

**Figure S7** Funnel plot of the meta-analysis in MDD.

**Figure S8** Funnel plot of the meta-analysis in BD.

**Figure S9** Funnel plot of the meta-analysis in SZ.

**REFERENCES**

**INCLUDED STUDIES**

**SUPPLEMENTARY METHODS**

**I. Quality assessment checklist**

To indicate the methodological limitations of published studies and weight the importance of these findings, Imaging Methodology Quality Assessment Checklist was used to objectively assess the integrity of included articles, which was a 12-point checklist with three categories: subjects (items 1-4), methods for image acquisition and analysis (items 5-10), and results and conclusions (items 11-12). The items of checklist were revised to fit into the purposes of our meta-analysis and enhanced the transparency of reports. Each item receives a score of 1, 0.5 or 0 according to the criteria that were fully, partially or not met, respectively. The details are as below:

*Category 1: Subjects Score*

1. Patients were evaluated prospectively, specific diagnostic criteria were applied, and demographic data was reported.

2. Healthy control subjects were evaluated prospectively, psychiatric and medical illnesses were excluded, and demographic data were reported.

3. Important clinical variables (e.g. clinical scales, illness history, number of episodes; drug status, non-drug therapy status) were reported.

4. Sample size per group > 10, and no significant difference in age and sex existed.

*Category 2: Methods for image acquisition and analysis*

5. Magnet strength at least 1.5T.

6. Whole brain analysis was automated without priori regional selection.

7. Coordinates were reported in a standard space.

8. The imaging technique used was clearly described so as it could be reproduced.

9. Measurements were clearly described so that they could be reproduced.

10. Results have been corrected for multiple comparison.

*Category 3: Results and conclusions*

11. Statistical parameters for significant and important non-significant differences were provided.

12. Conclusions were consistent with the results obtained and the limitations were discussed.

**II. Neuroimaging meta-analysis implemented by AES-SDM**

All the neuroimaging meta-analyses in this study were performed using the Anisotropic Effect-Size Seed-based d Mapping (AES-SDM) software package (version 5.15) (1). AES-SDM estimates voxel-wise effect-size and variance maps by combining reported peak coordinates and statistical parameters from original studies, following the maximum-likelihood estimation approach of the MetaNSUE algorithm (2). Considering sample size, within-study variance, and between-study heterogeneity, random-effects models were applied to compute meta-analytical case-control differences. To enhance validity, AES-SDM implements Jackknife sensitivity analyses by iteratively repeating the meta-analysis while excluding one study at a time, assessing the stability of the results (3). All meta-analyses followed the guidelines provided in the AES-SDM online manual ([https://www.sdmproject.com/old/manual/](https://www.sdmproject.com/manual/)).

Specifically, we performed the following steps. First, we extracted peak coordinates and their corresponding t-values for each contrast from the included studies and created an AES-SDM table to record demographic and clinical information. For studies reporting no significant results, we included them with a note indicating the absence of peaks, and Z-values reported in the original studies were converted to t-values using the AES-SDM online converter. For studies lacking explicit uncorrected thresholds, we used a conservative t-value equivalent to P = 0.001. Second, peak coordinates and t-values were used to recreate study-specific effect-size and variance maps using an anisotropic Gaussian kernel. Third, a voxel-wise mean map of regional homogeneity (ReHo) differences was computed by weighting each study according to its sample size and variance within the random-effects framework. Larger studies with less variance contributed more to the mean maps. Finally, statistical significance was determined using a default empirical threshold recommended for AES-SDM (voxel-level *p* < 0.005, SDM-Z > 1, cluster extent ≥ 10 voxels).

**III. Heterogeneity, Jackknife Sensitivity, Publication Bias**

Between-study heterogeneity was assessed using a random-effects model with Q statistics, which evaluate unexplained variance across studies. Heterogeneity was quantified using the I² index, with permutation testing to estimate the proportion of total variance attributable to heterogeneity rather than chance. To evaluate the robustness of the meta-analytic findings, jackknife sensitivity analyses were performed within the AES-SDM framework by iteratively repeating the meta-analysis while excluding one study at a time. This leave-one-out procedure assesses whether significant clusters are driven by any single dataset and evaluates the stability of the results. Findings that remained significant across the majority of jackknife iterations were considered reliable. Second, potential publication bias was examined using Egger’s test and visual inspection of funnel plots. A p-value < 0.05 in Egger’s test, alongside a visibly asymmetric funnel plot, was considered indicative of bias (4,5).

**SUPPLEMENTARY RESULTS**

**I. Common and distinct ReHo abnormalities between MDD and BD**

The conjunction analysis revealed shared ReHo reduction in the left postcentral gyrus, right precuneus, and left lingual gyrus between MDD and BD. Compared with MDD, BD patients exhibited greater ReHo reduction in the left precuneus, left cerebellar hemispheric lobules, and left anterior cingulate/paracingulate cortex. Conversely, MDD showed decreased ReHo in the right temporal pole extending into the superior temporal gyrus compared with BD.

**II. Common and distinct ReHo abnormalities between BD and SZ**

The conjunction analysis demonstrated common ReHo reduction in the left precuneus and left medial superior orbitofrontal gyrus, as well as an increase in the right basal ganglia and insular regions between BD and SZ. Compared with BD, SZ patients exhibited increased ReHo in the left medial superior frontal gyrus, bilateral precuneus, and left precentral gyrus. In contrast, SZ patients showed greater ReHo reduction in the right insula relative to BD.

**III. Common and distinct ReHo abnormalities between MDD and SZ**

The conjunction analysis revealed shared ReHo increase in the rIFG and shared decreases in sensorimotor regions between MDD and SZ. Compared with MDD, SZ patients showed higher ReHo in the cingulate-striatal circuitry. In contrast, MDD patients exhibited relatively higher ReHo in parietal-occipital and posterior cingulate regions.

**IV. Heterogeneity, Jackknife Sensitivity, Publication Bias**

Between-study heterogeneity was generally low across most regions showing significant ReHo abnormalities in MDD, BD, or SZ. Specifically, I² values for these clusters were generally below 50%, indicating low inter-study heterogeneity (6). However, substantial heterogeneity was detected in the bilateral striatum (left: Q = 294.19, I² = 90.48%, *p* < 0.001; right: Q = 130.42, I² = 78.53%, *p* < 0.001) for SZ meta-analysis. In the jackknife sensitivity analysis, all significant clusters were preserved in more than 90% of the iterations, indicating high robustness and reproducibility of the findings (***Tables S11-13***). For BD meta-analysis, significant publication bias was detected in the precuneus and precentral gyrus (***Figure S8***). For SZ meta-analysis, significant publication bias was detected in the striatum, posterior cingulate cortex, occipital cortex and medial orbitofrontal cortex (***Figure S9***). This kind of bias is often assumed to be the case in neuroimaging meta-analyses with limited numbers of studies and substantial between-study variability (7).

| **Table S1** Demographic and clinical characteristics of included MDD studies. | | | | | | | | | | | | | |
| --- | --- | --- | --- | --- | --- | --- | --- | --- | --- | --- | --- | --- | --- |
| Study | MDD | HC | Age MDD | Age HC | Scanner | Software | Threshold | FWHM (mm) | Illness  duration (year) | Onset | Quality scores | Kendall’s W | Neighbor selection |
| Chen et al., 2012.a | 15 | 15 | 24.07 | 23.93 | 1.5T | SPM8 | P<0.05,AlphaSim | 8 | 0.28 | 24.07 | 12 | Yes | 27 |
| Chen et al., 2012.b | 15 | 15 | 67.53 | 64.87 | 1.5T | SPM8 | P<0.05,AlphaSim | 8 | 0.49 | 67.53 | 12 | Yes | 27 |
| Chen et al., 2023.a | 63 | 81 | 38.33 | 36.63 | 3.0T | DPABI  V5.1 | P<0.05,GRF | 6 | 2.66 | 35.75 | 11.75 | Yes | 27 |
| Chen et al., 2023.b | 33 | 36 | 45.24 | 44.75 | 3.0T | DPABI  V5.1 | P<0.05,GRF | 6 | 2.42 | 42.88 | 11.75 | Yes | 27 |
| Dai et al., 2024.a | 44 | 44 | 30.07 | 30.80 | 3.0T | DPARSF | P<0.05 | 4 | 1.13 | NA | 11.5 | Yes | 27 |
| Dai et al., 2024.b | 44 | 44 | 30.98 | 30.8 | 3.0T | DPARSF | P<0.05 | 4 | 1.13 | NA | 11.5 | Yes | 27 |
| Dan et al., 2023.a | 24 | 32 | 31.375 | 29.59 | 3.0T | DPARSF | P<0.05,GRF | 4 | 0.49 | 31.38 | 11.75 | Yes | 27 |
| Dan et al., 2023.b | 33 | 32 | 29.48 | 29.59 | 3.0T | DPARSF | P<0.05,GRF | 4 | 0.56 | 29.48 | 11.75 | Yes | 27 |
| Fang et al., 2022 | 42 | 20 | 28.85 | 27.7 | 3.0T | SPM8 | P<0.05,AlphaSim | 4 | 1.80 | 27.05 | 12 | Yes | 27 |
| Guo et al., 2012.a | 23 | 19 | 27.35 | 24.37 | 1.5T | SPM8 | P<0.05,Corrected | NA | 2.29 | 25.06 | 12 | NA | NA |
| Guo et al., 2012.b | 22 | 19 | 28.09 | 24.37 | 1.5T | SPM8 | P<0.05,Corrected | NA | 0.25 | 28.09 | 12 | NA | NA |
| Huang et al., 2014 | 23 | 20 | 27.7 | 28.8 | 3.0T | SPM5 | P<0.001,Corrected | 4 | NA | NA | 11 | Yes | 27 |
| Huang et al., 2024.a | 54 | 66 | 20.8 | 21.1 | 3.0T | DPABI | P<0.05 | 6 | 3.5 | 17.3 | 11.75 | NA | NA |
| Huang et al., 2024.b | 68 | 66 | 20.3 | 21.1 | 3.0T | DPABI | P<0.05 | 6 | 3.7 | 16.6 | 11.75 | NA | NA |
| Liang et al., 2013 | 16 | 16 | 36.06 | 34.47 | 3.0T | SPM8 | P<0.05,AlphaSim | 8 | 2.36 | 33.7 | 11.25 | Yes | 27 |
| Liang et al., 2020 | 67 | 69 | 35 | 34.1 | 1.5T | SPM12 | P<0.05,FWE | 8 | 1.85 | 33.15 | 11.75 | Yes | 27 |
| Li.G.Z et al., 2018 | 31 | 28 | 33.13 | 32.96 | 3.0T | SPM8 | P<0.05,GRF | 6 | 0.98 | 32.33 | 11.75 | NA | NA |
| Li.M et al., 2018 | 16 | 14 | 27.38 | 30.43 | 3.0T | SPM12 | P<0.05 | 4 | NA | NA | 10.75 | Yes | 27 |
| Lin et al., 2022 | 34 | 30 | 37.74 | 35.8 | 3.0T | SPM8 | P<0.001,FDR | NA | 5.44 | 32.3 | 12 | Yes | 27 |
| Liu et al., 2021.a | 24 | 70 | 28.33 | 31.75 | 3.0T | DPABI 4.3 | P<0.05 | 6 | 0.50 | 28.33 | 11.75 | Yes | 27 |
| Liu et al., 2021.b | 50 | 70 | 33.33 | 31.76 | 3.0T | DPABI 4.3 | P<0.05 | 6 | 0.43 | 33.33 | 11.75 | Yes | 27 |
| Liu et al., 2021 | 35 | 19 | 26.17 | 28.16 | 3.0T | DPABI 1.3 | P<0.05 | 4 | 2 | 24.17 | 11.25 | Yes | NA |
| Liu et al., 2017 | 23 | 24 | 37.78 | 38.71 | 3.0T | SPM8 | P<0.01 | 4 | NA | NA | 11.75 | Yes | 27 |
| Liu et al., 2010 | 15 | 15 | 29.13 | 30.20 | 3.0T | SPM2 | P<0.001,Uncorrected | 4 | 1.1 | 28.03 | 11 | Yes | 27 |
| Ni et al., 2023 | 60 | 60 | 29.05 | 29.10 | 3.0T | DPABI | P<0.05 | 4 | 2.95 | 26.1 | 12 | Yes | 27 |
| Ni et al., 2024 | 63 | 45 | 27.59 | 28.20 | 3.0T | SPM12 | P<0.05,Bonferroni | 4 | 2.49 | 25.1 | 11.75 | Yes | 27 |
| Peng et al., 2011 | 16 | 16 | 34.1 | 33.7 | 3.0T | SPM2 | P<0.01,AlphaSim | 6 | 0.26 | 34.1 | 11.5 | Yes | 27 |
| Shen et al., 2017.a | 58 | 60 | 23.98 | 24.63 | 3.0T | DPARSF | P<0.05,FDR | 4 | 0.75 | 23.23 | 11.75 | Yes | 27 |
| Shen et al., 2017.b | 62 | 52 | 36.74 | 36.83 | 3.0T | DPARSF | P<0.05,FDR | 4 | 0.69 | 36.06 | 11.75 | Yes | 27 |
| Song et al., 2016 | 31 | 31 | 33.8 | 34.7 | 3.0T | SPM8 | P<0.05,AlphaSim | 6 | 2.84 | 30.96 | 11.75 | Yes | 27 |
| Song et al., 2020 | 15 | 15 | 47.15 | 42.27 | 3.0T | SPM8 | P<0.05,Corrected | 4 | NA | NA | 11.5 | NA | NA |
| Sun et al., 2022 | 36 | 36 | 31.58 | 31.61 | 3.0T | DPABI 5.0 | P<0.05 | 6 | 1.79 | 29.79 | 12 | Yes | 27 |
| Tu et al., 2022 | 77 | 78 | 29.76 | 28.45 | 3.0T | SPM12 | P<0.05,GRF | 6 | 0.85 | 29.76 | 11.75 | Yes | 27 |
| Wang et al., 2013 | 14 | 14 | 32.93 | 34.14 | 3.0T | DPARSF | P<0.05,AlphaSim | 4 | 0.51 | 32.93 | 12 | Yes | 27 |
| Wu et al., 2011 | 44 | 26 | 33 | 35 | 3.0T | SPM5 | P<0.05,FDR | NA | 5.63 | 29.37 | 11 | Yes | 27 |
| Wu et al., 2024 | 55 | 57 | 42.27 | 48.11 | 3.0T | DPARSF 5.2 | P<0.05 | 6 | NA | NA | 11.5 | Yes | 27 |
| Yang et al., 2015 | 50 | 50 | 31.12 | 31.3 | 3.0T | SPM8 | P<0.001 | 6 | 0.82 | 31.12 | 11.75 | Yes | 27 |
| Zhang.A.X et al., 2022 | 101 | 53 | 34.5 | 35.92 | 3.0T | SPM8 | P<0.05,AlphaSim | 6 | NA | NA | 11.5 | NA | NA |
| Zhang.B et al., 2022 | 36 | 33 | 20.83 | 19.18 | 3.0T | SPM8 | P<0.01 | NA | NA | NA | 11.5 | Yes | 27 |
| Zhang et al., 2021 | 91 | 115 | 30.55 | 31.4 | 3.0T | SPM8 | P<0.05 | 6 | 1.78 | 28.9 | 12 | Yes | 27 |
| Zhao et al., 2023 | 30 | 30 | 31.2 | 30.2 | 3.0T | SPM12 | P<0.05,FDR | 6 | 0.5 | 31.2 | 11.75 | Yes | 27 |
| Zhou et al., 2022.a | 25 | 25 | 14.48 | 14.96 | 3.0T | SPM12 | P<0.05,Bonferroni | 4 | 1.67 | 12.72 | 11.5 | NA | NA |
| Zhou et al., 2022.b | 25 | 25 | 14.96 | 14.96 | 3.0T | SPM12 | P<0.05,Bonferroni | 4 | 0.68 | 14.28 | 11.5 | NA | NA |

***Abbreviations:*** MDD = Major Depressive Disorder; HC = Healthy Control; ReHo = Regional Homogeneity; SPM = Statistical Parametric Mapping; FWE = Family Wise Error; FDR = False Discovery Rate; GRF = Gaussian Random Field; NA = Not Available.

| **Table S2** Demographic and clinical characteristics of included BD studies. | | | | | | | | | | | | | |
| --- | --- | --- | --- | --- | --- | --- | --- | --- | --- | --- | --- | --- | --- |
| Study | BD | HC | Age BD | Age HC | Scanner | Software | Threshold | FWHM （mm） | Illness  duration (year) | Onset year | Quality scores | Kendall’s W | Neighbor selection |
| Adrian et al., 2021 | 19 | 18 | 36 | 35 | 3.0T | SPM12 | P<0.001 | NA | NA | 22.5 | 12 | Yes | 27 |
| Cui et al., 2016 | 19 | 18 | 15.05 | 14.06 | 3.0T | DPARSF  V3.1 | P<0.01 | 8 | 1.16 | 13.84 | 12 | Yes | 27 |
| Gao et al., 2014 | 17 | 18 | 14.4 | 14.1 | 3.0T | SPM8 | P<0.05 | 4 | 0.88 | 13.5 | 12 | Yes | 27 |
| Jiang et al., 2016 | 24 | 28 | 26.88 | 26.54 | 3.0T | SPM8 | P<0.001 | 6 | NA | NA | 11.75 | NA | NA |
| Lei et al., 2024 | 56 | 71 | 27 | 26 | 3.0T | DPABI  V3.1 | P<0.05 | 4 | 3.82 | 52.18 | 11.25 | Yes | 27 |
| Liang et al., 2013 | 17 | 16 | 34.47 | 35.13 | 1.5T | DPARSF | P<0.05 | 8 | 3.89 | 30.58 | 10.75 | Yes | 27 |
| Li et al., 2020 | 44 | 36 | 23.11 | 22.78 | 3.0T | SPM12 | P<0.05 | 6 | 1.81 | 21.3 | 11.75 | Yes | 27 |
| Liu et al., 2012 | 26 | 26 | 32.35 | 31.92 | 3.0T | SPM8 | P<0.01 | 4 | 4.20 | 28.15 | 11.75 | Yes | 27 |
| Liu et al., 2020 | 40 | 54 | 32.85 | 32.61 | 3.0T | DPABI 2.2 | P<0.01 | 6 | NA | NA | 11.5 | NA | NA |
| Ni et al., 2024 | 51 | 45 | 25.10 | 28.2 | 3.0T | SPM12 | P<0.05 | 4 | 3.18 | 21.92 | 11.75 | Yes | 27 |
| Qiu et al., 2019 | 100 | 100 | 26.37 | 28.32 | 3.0T | SPM12 | P<0.05 | 4 | 4.44 | 21.93 | 11.75 | Yes | 27 |
| Russo et al., 2020 | 216 | 112 | 46.04 | 36.74 | 3.0T | AFNI | P<0.001 | 6 | NA | NA | 9.5 | Yes | 27 |
| Shan et al., 2020 | 37 | 37 | 20.97 | 20.84 | 3.0T | REST | P<0.05 | 4 | NA | NA | 11.75 | Yes | NA |
| Wu et al., 2023 | 37 | 37 | 17.59 | 21.14 | 3.0T | DPARSF | P<0.05 | 6 | 1 | 16.59 | 11.75 | NA | NA |
| Xiao et al., 2013 | 15 | 15 | 15 | 14.1 | 3.0T | SPM8 | P<0.05 | 4 | NA | NA | 11.5 | Yes | 27 |
| Xiao et al., 2019 | 43 | 19 | 15.01 | 14.16 | 3.0T | SPM8 | P<0.01 | 4 | 3.0 | 14.10 | 12 | Yes | 27 |
| Yao et al., 2018 | 55 | 113 | 27.02 | 28.96 | 3.0T | SPM8 | P<0.005 | 6 | 3.87 | 23.15 | 12 | Yes | 27 |
| Zhao et al., 2024 | 56 | 71 | 27 | 26 | 3.0T | DPABI V3.1. | P<0.05 | 4 | 3.82 | 23.18 | 11.75 | Yes | 27 |

***Abbreviations:*** BD = Bipolar Disorder; HC = Healthy Control; ReHo = Regional Homogeneity; SPM = Statistical Parametric Mapping; NA = Not Available; REST = Resting-State fMRI Data Analysis Toolkit; DPARSF = Data Processing Assistant for Resting-State fMRI; DPAB I = Data Processing & Analysis for Brain Imaging ; NA = Not Available.

| **Table S3** Demographic and clinical characteristics of included SZ studies. | | | | | | | | | | | | | |
| --- | --- | --- | --- | --- | --- | --- | --- | --- | --- | --- | --- | --- | --- |
| Study | SZ | HC | Age SZ | Age HC | Scanner | Software | Threshold | FWHM （mm） | Illness  duration (year) | Onset year | Quality scores | Kendall’s W | Neighbor selection |
| Chen et al., 2025 | 23 | 28 | 34.91 | 39.29 | 3.0T | DPARSFA  V5.4 | P<0.05 | 4 | 12.74 | 22.17 | 11.5 | Yes | 27 |
| Cui et al., 2016 | 17 | 19 | 21.24 | 23.79 | 3.0T | DPARSFA V3.1 | P<0.01 | 4 | 0.54 | NA | 10.75 | Yes | 27 |
| Cui et al., 2016 | 15 | 19 | 22.53 | 23.79 | 3.0T | DPARSFA V3.1 | P<0.01 | 4 | 0.85 | NA | 10.75 | Yes | 27 |
| Fang et al., 2013 | 13 | 13 | 25 | 25.83 | 3.0T | SPM8 | P<0.05 | 4 | 1.13 | 23.87 | 11.5 | Yes | 27 |
| Gao et al., 2015 | 14 | 14 | 33.2 | 34.9 | 1.5T | DPARSF | P<0.001 | 6 | 9.2 | 24 | 12 | Yes | 27 |
| Gao et al., 2018.a | 17 | 29 | 31.24 | 32.73 | 3.0T | SPM8 | P<0.001 | 4 | 14 | 17.24 | 12 | Yes | 27 |
| Gao et al., 2018.b | 17 | 29 | 36.82 | 32.73 | 3.0T | SPM8 | P<0.001 | 4 | 7.88 | 29.18 | 12 | Yes | 27 |
| Gao et al., 2020 | 57 | 50 | 31.63 | 28.38 | 3.0T | SPM12 | P<0.05 | 4 | 2.52 | 29.11 | 12 | Yes | 27 |
| Gou et al., 2018 | 28 | 21 | 23.9 | 28.6 | 1.5T | DPARSF | P<0.05 | 4 | 1.26 | 22.64 | 11.75 | NA | NA |
| Huang et al., 2022 | 187 | 100 | 25.25 | 25.29 | 3.0T | SPM12 | P<0.05,Uncorrected | 4 | 1.10 | 24.15 | 11.25 | NA | NA |
| Hu et al., 2016 | 42 | 38 | 24.86 | 24.76 | 3.0T | DPARSF | P<0.05 | NA | 0.70 | 24.16 | 12 | Yes | 27 |
| Jiang et al., 2010 | 18 | 18 | 16.3 | 16.4 | 1.5T | SPM5 | P<0.05 | 4 | 0.8 | 15.4 | 10.5 | Yes | 27 |
| Jin et al., 2021 | 23 | 24 | 31.74 | 30.92 | 3.0T | SPM8 | P<0.05 | 6 | NA | NA | 11.75 | Yes | NA |
| Li et al., 2024 | 21 | 21 | 16.62 | 16.81 | 3.0T | REST | P<0.001 | 6 | 0.48 | 16.14 | 12 | Yes | 27 |
| Liu et al., 2016 | 27 | 27 | 25.44 | 27.44 | 1.5T | SPM8 | P<0.05 | NA | 1.53 | 23.91 | 12 | Yes | 27 |
| Liu et al., 2006 | 18 | 18 | 23.67 | 24.44 | 1.5T | SPM2 | P<0.001 | 4 | NA | NA | 11.25 | Yes | 27 |
| Liu et al., 2025 | 56 | 56 | 28.29 | 27.71 | 3.0T | DPARSF V2.3 | P<0.05 | 6 | NA | NA | 11.75 | Yes | 27 |
| Shan et al., 2021 | 39 | 20 | 24.4 | 25.7 | 3.0T | DPABI  V4.2 | P<0.05,FDR | 4 | NA | NA | 11.75 | Yes | 27 |
| Wang et al., 2017 | 48 | 31 | 15.79 | 15.42 | 3.0T | REST | P<0.05,Uncorrected | 4 | 0.45 | 15.79 | 11.5 | Yes | 27 |
| Wu et al., 2018 | 43 | 56 | 26.42 | 25.07 | 3.0T | SPM8 | P<0.05 | 8 | NA | NA | 10.75 | Yes | 27 |
| Yan et al., 2020 | 69 | 74 | 24.22 | 26.27 | 3.0T | DPARSF  V4.4 | P<0.05 | 4 | NA | NA | 11.75 | Yes | 27 |
| Yu et al., 2013 | 69 | 62 | 31.7 | 29.9 | 3.0T | SPM5 | P<0.05 | 6 | 7.1 | 24.6 | 11.5 | Yes | 27 |
| Zhang et al., 2024 | 58 | 65 | 28.41 | 27.48 | 3.0T | DPARSF V5.4 | P<0.05 | 4 | 5.54 | 22.87 | 12 | Yes | 27 |
| Zhang.R.R et al., 2018 | 28 | 38 | 37.57 | 51.79 | 3.0T | DPABI | P<0.05 | 4 | 1.04 | 20.86 | 11.75 | Yes | 27 |
| Zhang.S.Y et al., 2018 | 16 | 18 | 23.8 | 23.2 | 3.0T | SPM8 | P<0.001 | 4 | NA | NA | 11.5 | Yes | 27 |
| Zhang et al., 2017 | 22 | 23 | 28 | 28 | 3.0T | SPM12 | P<0.05 | 4 | NA | NA | 10.75 | Yes | NA |
| Zhang et al., 2019 | 53 | 67 | 36.75 | 34.82 | 3.0T | DPABI | P<0.05,FDR | 6 | 14.94 | 21.81 | 11.25 | Yes | 27 |
| Zhao et al., 2019 | 44 | 26 | 23.7 | 22.6 | 3.0T | DPARSF | P<0.05 | 4 | NA | NA | 12 | Yes | 27 |
| Zhou et al., 2024 | 107 | 67 | 15.33 | 15.43 | 3.0T | SPM12 | P<0.05 | NA | 0.46 | 15.33 | 12 | Yes | 27 |

***Abbreviations:*** SZ = Schizophrenia; HC = Healthy Control; ReHo = Regional Homogeneity; FDR = False Discovery Rate; SPM = Statistical Parametric Mapping; REST = Resting-State fMRI Data Analysis Toolkit; DPARSF = Data Processing Assistant for Resting-State fMRI; DPABI = Data Processing & Analysis for Brain Imaging; NA = Not Available.

| **Table S4** Meta-analytic findings on ReHo abnormalities in MDD. | | | | | |
| --- | --- | --- | --- | --- | --- |
| Effect anatomy/region | MNI peak coordinates  X,Y,Z | Voxels | SDM-Z value | P value | Breakdown  (No. of voxels) |
| ***MDD > HC*** | | | | | |
| Right superior frontal gyrus, medial/Right anterior cingulate / paracingulate gyri | 10,46,8 | 629 | 1.813 | 0.000092924 | Right superior frontal gyrus, medial (160) |
|  |  |  |  |  | Right anterior cingulate / paracingulate gyri(93) |
|  |  |  |  |  | Right superior frontal gyrus, medial orbital(83) |
|  |  |  |  |  | Left anterior cingulate / paracingulate gyri(77) |
|  |  |  |  |  | Left anterior cingulate / paracingulate gyri(55) |
|  |  |  |  |  | Right anterior cingulate / paracingulate gyri(43) |
|  |  |  |  |  | Left superior frontal gyrus, medial(30) |
|  |  |  |  |  | Right superior frontal gyrus, medial orbital(21) |
|  |  |  |  |  | Left superior frontal gyrus, medial orbital(21) |
|  |  |  |  |  | Left superior frontal gyrus, medial(18) |
|  |  |  |  |  | Left anterior cingulate / paracingulate gyri(18) |
|  |  |  |  |  | Left superior frontal gyrus, medial orbital(10) |
| Right middle temporal gyrus | 40,-66,24 | 127 | 1.499 | 0.000655413 | Right middle temporal gyrus(67) |
|  |  |  |  |  | Right middle occipital gyrus(26) |
|  |  |  |  |  | Right middle temporal gyrus(18) |
|  |  |  |  |  | Right angular gyrus(16) |
| Left hippocampus | -28,-26,-16 | 57 | 1.358 | 0.001321197 | Left hippocampus(45) |
|  |  |  |  |  | Left parahippocampal gyrus(12) |
| Right inferior frontal gyrus | 46,18,16 | 30 | 1.184 | 0.003142953 | Right inferior frontal gyrus, triangular part(18) |
|  |  |  |  |  | Right inferior frontal gyrus, opercular part(12) |
| ***MDD < HC*** | | | | | |
| Left sensorimotor cortex/Left insular cortex | -56,-2,26 | 832 | -2.126 | 0.000190973 | Left insula(202) |
|  |  |  |  |  | Left postcentral gyrus(171) |
|  |  |  |  |  | Left rolandic operculum(98) |
|  |  |  |  |  | Left lenticular nucleus, putamen, BA 48(74) |
|  |  |  |  |  | Left precentral gyrus, BA 4(52) |
|  |  |  |  |  | Left precentral gyrus, BA 6(46) |
|  |  |  |  |  | Left postcentral gyrus, BA 3(46) |
|  |  |  |  |  | Left postcentral gyrus, BA 48(45) |
|  |  |  |  |  | Left postcentral gyrus, BA 4(33) |
|  |  |  |  |  | Left lenticular nucleus, putamen(32) |
|  |  |  |  |  | Left inferior frontal gyrus, opercular part, BA 6(18) |
|  |  |  |  |  | Left striatum(15) |
| Bilateral occipital visual cortex | 0,-82,18 | 389 | -2.130 | 0.000185788 | Left cuneus cortex, BA 18(149) |
|  |  |  |  |  | Right cuneus cortex, BA 18(89) |
|  |  |  |  |  | Left calcarine fissure / surrounding cortex, BA 17(64) |
|  |  |  |  |  | Left calcarine fissure / surrounding cortex, BA 18(60) |
|  |  |  |  |  | Left cuneus cortex(27) |
| Right striatum/Right lenticular nucleus, putamen | 26,14,-2 | 215 | -1.971 | 0.000418007 | Right lenticular nucleus, putamen(105) |
|  |  |  |  |  | Right striatum(99) |
|  |  |  |  |  | Left middle occipital gyrus, BA 19(11) |

***Abbreviations:*** MDD = Major Depressive Disorder; HC = Healthy Control; ReHo = Regional Homogeneity.

**Table S5** Meta-analytic findings on ReHo abnormalities in BD.

| Effect anatomy/region | MNI peak coordinates  X,Y,Z | Voxels | SDM-Z value | Maximum P (density statistic) | Breakdown  (No. of voxels) |
| --- | --- | --- | --- | --- | --- |
| ***BD > HC*** | | | | | |
| Right insula/Right temporal pole, superior temporal gyrus/Right inferior frontal gyrus, orbital part | 42,10,-12 | 782 | 2.208 | 0.000098050 | Right temporal pole, superior temporal gyrus, BA 38(298) |
|  |  |  |  |  | Right insula, BA 48(265) |
|  |  |  |  |  | Right temporal pole, superior temporal gyrus(74) |
|  |  |  |  |  | Right insula(48) |
|  |  |  |  |  | Right insula, BA 38(40) |
|  |  |  |  |  | Right inferior frontal gyrus, orbital part, BA 38(38) |
|  |  |  |  |  | Right temporal pole, superior temporal gyrus, BA 21(19) |
|  |  |  |  |  | Right superior temporal gyrus, BA 48(19) |
|  |  |  |  |  | Right temporal pole, middle temporal gyrus, BA 38(17) |
|  |  |  |  |  | Right insula, BA 47(16) |
|  |  |  |  |  | Right temporal pole, superior temporal gyrus, BA 48(16) |
|  |  |  |  |  | Left striatum(17) |
|  |  |  |  |  | Right striatum(10) |
| Left inferior frontal gyrus, orbital part | -46,28,-10 | 119 | 1.787 | 0.001414061 | Left inferior frontal gyrus, orbital part, BA 47 (119) |
| ***BD < HC*** | | | | | |
| Left precuneus | -4,-62,26 | 552 | -2.788 | ~0 | Left precuneus(204) |
|  |  |  |  |  | Right precuneus(159) |
|  |  |  |  |  | Left precuneus, BA 23(82) |
|  |  |  |  |  | Right precuneus, BA 23(61) |
|  |  |  |  |  | Left precuneus, BA 30(23) |
|  |  |  |  |  | Right precuneus, BA 7(23) |
| Cerebellum, vermic lobule IV / V | 0,-56,-10 | 165 | -2.241 | 0.000392199 | Cerebellum, vermic lobule IV / V(79) |
|  |  |  |  |  | Left cerebellum, hemispheric lobule IV / V, BA 18(38) |
|  |  |  |  |  | Cerebellum, vermic lobule IV / V, BA 18(28) |
|  |  |  |  |  | Left lingual gyrus, BA 18(20) |
| Left precentral gyrus, | -58,-2,30 | 75 | -1.902 | 0.002172709 | Left precentral gyrus, BA 6(43) |
|  |  |  |  |  | Left inferior frontal gyrus, opercular part, BA 44(20) |
|  |  |  |  |  | Left precentral gyrus, BA 4(12) |

***Abbreviations:*** BD = Bipolar Disorder; HC = Healthy Control; ReHo = Regional Homogeneity**.**

**Table S6** Meta-analytic findings on ReHo abnormalities in SZ.

| Effect anatomy/region | MNI peak coordinates  X,Y,Z | Voxels | SDM-Z value | Maximum P (density statistic) | Breakdown  (No. of voxels) |
| --- | --- | --- | --- | --- | --- |
| ***SZ > HC*** | | | | | |
| Left striatum/Left anterior thalamic projections/Right anterior thalamic projections/Right inferior frontal gyrus, triangular part | -22,14,-8 | 2083 | 2.745 | 0.000010312 | Right striatum(422) |
|  |  |  |  |  | Right inferior frontal gyrus, triangular part, BA 45(313) |
|  |  |  |  |  | Right lenticular nucleus, putamen, BA 48(288) |
|  |  |  |  |  | Left striatum(264) |
|  |  |  |  |  | Left lenticular nucleus, putamen, BA 48(176) |
|  |  |  |  |  | Right inferior frontal gyrus, orbital part, BA 47(168) |
|  |  |  |  |  | Left caudate nucleus(120) |
|  |  |  |  |  | Left insula, BA 48(95) |
|  |  |  |  |  | Right amygdala, BA 34(56) |
|  |  |  |  |  | Left amygdala, BA 34(49) |
|  |  |  |  |  | Left lenticular nucleus, putamen(45) |
|  |  |  |  |  | Left olfactory cortex, BA 48(37) |
|  |  |  |  |  | Right caudate nucleus, BA 25(30) |
| Medial prefrontal cortex | 10,26,40 | 1260 | 2.725 | 0.000010312 | Left superior frontal gyrus, medial, BA 8(223) |
|  |  |  |  |  | Left superior frontal gyrus, medial, BA 9(182) |
|  |  |  |  |  | Left superior frontal gyrus, medial, BA 32(129) |
|  |  |  |  |  | Right superior frontal gyrus, medial, BA 8(127) |
|  |  |  |  |  | Right superior frontal gyrus, medial, BA 9(114) |
|  |  |  |  |  | Left superior frontal gyrus, medial(96) |
|  |  |  |  |  | Left median cingulate / paracingulate gyri, BA 24(79) |
|  |  |  |  |  | Right median cingulate / paracingulate gyri, BA 24(64) |
|  |  |  |  |  | Right superior frontal gyrus, medial, BA 32(58) |
|  |  |  |  |  | Left supplementary motor area, BA 8(54) |
|  |  |  |  |  | Right superior frontal gyrus, dorsolateral, BA 9(42) |
|  |  |  |  |  | Left anterior cingulate / paracingulate gyri, BA 24(25) |
|  |  |  |  |  | Left supplementary motor area, BA 32(24) |
|  |  |  |  |  | Right supplementary motor area, BA 8(23) |
|  |  |  |  |  | Left anterior cingulate / paracingulate gyri(20) |
| Left angular gyrus | -48,-60,38 | 124 | 1.972 | 0.000696719 | Left angular gyrus, BA 39(98) |
|  |  |  |  |  | Left inferior parietal (excluding supramarginal and angular) gyri(26) |
| ***SZ < HC*** | | | | | |
| Right precentral gyrus,/Right postcentral gyrus/Somatomotor areas | 54,0,38 | 894 | -2.480 | 0.000015497 | Right precentral gyrus, BA 6(354) |
|  |  |  |  |  | Right postcentral gyrus, BA 4(150) |
|  |  |  |  |  | Right precentral gyrus, BA 4(136) |
|  |  |  |  |  | Right postcentral gyrus, BA 43(101) |
|  |  |  |  |  | Right postcentral gyrus, BA 3(97) |
|  |  |  |  |  | Corpus callosum(56) |
| Bilateral posterior cingulate cortex/Left precuneus | 4,-40,26 | 305 | -1.761 | 0.000923812 | Left posterior cingulate gyrus, BA 23(79) |
|  |  |  |  |  | Left posterior cingulate gyrus (43) |
|  |  |  |  |  | Right median cingulate / paracingulate gyri, BA 23(42) |
|  |  |  |  |  | Left precuneus, BA 23(36) |
|  |  |  |  |  | Right posterior cingulate gyrus, BA 23 (33) |
|  |  |  |  |  | Right posterior cingulate gyrus, BA 26(31) |
|  |  |  |  |  | Left posterior cingulate gyrus, BA 26(21) |
|  |  |  |  |  | Right median network, cingulum (20) |
| Right occipital visual cortex | 30,-86,32 | 271 | -2.210 | 0.000092924 | Right superior occipital gyrus, BA 19(130) |
|  |  |  |  |  | Right cuneus cortex, BA 19(51) |
|  |  |  |  |  | Corpus callosum(38) |
|  |  |  |  |  | Right middle occipital gyrus, BA 19(33) |
|  |  |  |  |  | Right superior occipital gyrus, BA 18(19) |
| Left occipital visual cortex/Left superior occipital gyrus | -12,-100,16 | 153 | -2.037 | 0.000263214 | Left superior occipital gyrus, BA 17(88) |
|  |  |  |  |  | Left middle occipital gyrus, BA 17(33) |
|  |  |  |  |  | Left superior occipital gyrus, BA 18(17) |
|  |  |  |  |  | Left cuneus cortex, BA 17(15) |
| Right occipital visual cortex | 34,-94,0 | 164 | -2.091 | 0.000185788 | Right middle occipital gyrus, BA 18(100) |
|  |  |  |  |  | Right inferior occipital gyrus, BA 18(47) |
|  |  |  |  |  | Right inferior occipital gyrus, BA 19(17) |
| Left medial orbitofrontal | -6,26,-6 | 60 | -2.153 | 0.000149667 | Left superior frontal gyrus, medial orbital, BA 11(25) |
|  |  |  |  |  | Left anterior cingulate / paracingulate gyri, BA 11(14) |
|  |  |  |  |  | Left olfactory cortex, BA 11(11) |
|  |  |  |  |  | Left olfactory cortex, BA 25(10) |

***Abbreviations:*** SZ = Schizophrenia; HC = Healthy Control; ReHo = Regional Homogeneity**.**

**Table S7** Meta-regression findings.

| Disorder | Demographic Factor | Regions | Peak coordinates  (x,y,z) | SDM-Z | Voxels | *P* value |
| --- | --- | --- | --- | --- | --- | --- |
| MDD | Age (+) | Right middle temporal gyrus | 52,-48,8 | 1.617 | 74 | < 0.001 |
|  | Age (–) | Left middle occipital gyrus | -38,-86,4 | -3.512 | 650 | < 0.001 |
|  |  | Right middle occipital gyrus | 44,-78,18 | -3.529 | 491 | < 0.001 |
|  |  | Right postcentral gyrus | 48,-20,48 | -2.646 | 394 | < 0.001 |
|  |  | Left cuneus cortex | 2,-74,22 | -2.368 | 81 | < 0.001 |
|  | Illness duration (-) | Right superior frontal gyrus, medial orbital | 2,42,-10 | -2.602 | 142 | < 0.001 |
|  | Onset age (-) | Left middle occipital gyrus | -38,-86,4 | -3.381 | 1071 | < 0.001 |
| SZ | Age (–) | Left middle occipital gyrus | -46,-72,6 | -1.891 | 249 | < 0.001 |
|  | Illness duration (-) | Left middle occipital gyrus | -46,-72,4 | -2.962 | 466 | < 0.001 |
|  |  | Right superior parietal gyrus | 18,-60,60 | -2.022 | 88 | < 0.001 |

***Abbreviations:*** MDD = Major Depressive Disorder; SZ = Schizophrenia.

**Table S8** Common and distinct patterns of ReHo abnormalities between MDD and BD.

| Region | MNI peak coordinates  X,Y,Z | Voxels | Breakdown  (No. of voxels) |
| --- | --- | --- | --- |
| ***MDD & BD < HC*** | | | |
| Left postcentral gyrus | -60,-8,30 | 529 | Left postcentral gyrus (357)  Left precentral gyrus (79)  Left inferior frontal gyrus, opercular part (68)  Left rolandic operculum (25) |
| Right precuneus | 4,-68,26 | 133 | Right precuneus (79)  Left cuneus cortex (34)  Left precuneus (20) |
| Left median network, cingulum/Left lingual gyrus | -14,-50,0 | 115 | Left lingual gyrus (101)  Left median network, cingulum(14) |
| ***BD < MDD*** |  |  |  |
| Left precuneus | 0,-56,36 | 580 | Left precuneus (291)  Right precuneus (184)  Left posterior cingulate gyrus (46)  Left median network, cingulum (35)  Right median cingulate / paracingulate gyri (24) |
| Left cerebellum, hemispheric lobule IV / V | -4,-52,-4 | 50 | Cerebellum, vermic lobule IV / V (28)  Left cerebellum, hemispheric lobule IV / V (22) |
| Left anterior cingulate/paracingulate cortex | -12,42,0 | 47 | Left anterior cingulate / paracingulate gyri (32)  Left median network, cingulum(15) |
| ***BD > MDD*** |  |  |  |
| Right temporal pole, superior temporal gyrus | 46,22,-22 | 104 | Right temporal pole, superior temporal gyrus (82)  Right inferior frontal gyrus, orbital part (22) |

***Abbreviations:*** ReHo = Regional Homogeneity; MDD = Major depressive disorder; BD = Bipolar Disorder.

**Table S9** Common and distinct patterns of ReHo abnormalities between BD and SZ.

| Region | MNI peak coordinates  X,Y,Z | Voxels | Breakdown  (No. of voxels) |
| --- | --- | --- | --- |
| ***BD & SZ < HC*** | | | |
| Left precuneus | -2,-62,22 | 1359 | Left precuneus (363)  Left median network, cingulum (180)  Right median cingulate / paracingulate gyri (121)  Right precuneus (251)  Right median network, cingulum (109)  Left posterior cingulate gyrus (182)  Right posterior cingulate gyrus (85)  Left median cingulate / paracingulate gyri(39)  Left cuneus cortex(29) |
| ***BD & SZ > HC*** | | | |
| Right lenticular nucleus, putamen | 36,-2,-12 | 1025 | Right lenticular nucleus, putamen(403)  Right insula (228)  Right temporal pole, superior temporal gyrus(149)  Right striatum(106)  Right inferior frontal gyrus, orbital part (118)  Right amygdala (21) |
| Left striatum | -14,-4,-8 | 30 | Left striatum (30) |
| ***SZ > BD*** | | | |
| Left superior frontal gyrus, medial | -6,28,46 | 246 | Left superior frontal gyrus, medial(114)  Left superior frontal gyrus, medial (52)  Left supplementary motor area(34)  Left median cingulate / paracingulate gyri(26)  Right median cingulate / paracingulate gyri (20) |
| Left precuneus | 2,-56,40 | 202 | Left precuneus (108)  Right precuneus(94) |
| Left precentral gyrus | -52,10,30 | 79 | Left precentral gyrus(59)  Left inferior frontal gyrus, opercular part (20) |
| Left cerebellum, hemispheric lobule IV / V | -6,-52,-6 | 27 | Left cerebellum, hemispheric lobule IV / V(27) |
| ***SZ < BD*** | | | |
| Right insula | 32,12,-16 | 53 | Right temporal pole, superior temporal gyrus (43)  Right insula(10) |

***Abbreviations:*** ReHo = Regional Homogeneity; BD = Bipolar Disorder; SZ = Schizophrenia.

**Table S10** Common and distinct patterns of ReHo abnormalities between MDD and SZ.

| Region | MNI peak coordinates  X,Y,Z | Voxels | Breakdown  (No. of voxels) |
| --- | --- | --- | --- |
| ***MDD & SZ < HC*** | | | |
| Right precentral gyrus | 52,-10,42 | 599 | Right precentral gyrus, BA 6(205) |
|  |  |  | Right precentral gyrus, BA 4(140) |
|  |  |  | Right postcentral gyrus, BA 3(107) |
|  |  |  | Right postcentral gyrus, BA 4(96) |
|  |  |  | Right postcentral gyrus, BA 43(51) |
| Left postcentral gyrus | -62,-12,28 | 469 | Left postcentral gyrus, BA 43(234) |
|  |  |  | Left postcentral gyrus, BA 48(73) |
|  |  |  | Left precentral gyrus, BA 4(47) |
|  |  |  | Left postcentral gyrus, BA 3(46) |
|  |  |  | Left arcuate network, anterior segment(42) |
|  |  |  | Left postcentral gyrus, BA 4(27) |
| Left middle occipital gyrus | -38,-78,0 | 452 | Left middle occipital gyrus, BA 19(235) |
|  |  |  | Left inferior occipital gyrus, BA 19(131) |
|  |  |  | Left middle occipital gyrus, BA 37(59) |
|  |  |  | Left middle occipital gyrus, BA 18(17) |
|  |  |  | Left middle temporal gyrus, BA 37(10) |
| ***MDD & SZ > HC*** | | | |
| Right inferior frontal gyrus, triangular part | 50,24,18 | 567 | Right inferior frontal gyrus, triangular part, BA 45(354) |
|  |  |  | Right inferior frontal gyrus, opercular part, BA 48(119) |
|  |  |  | Right inferior frontal gyrus, triangular part, BA 48(57) |
|  |  |  | Right inferior frontal gyrus, opercular part, BA 45(21) |
|  |  |  | Right inferior frontal gyrus, opercular part, BA 44(16) |
| ***MDD > SZ*** | | | |
| Right postcentral gyrus | 52,-4,32 | 243 | Right postcentral gyrus, BA 4(111) |
|  |  |  | Right precentral gyrus, BA 6(78) |
|  |  |  | Right precentral gyrus, BA 4(44) |
|  |  |  | Right postcentral gyrus, BA 3(10) |
| Left posterior cingulate gyrus | -4,-50,20 | 27 | Left posterior cingulate gyrus, BA 30(17) |
|  |  |  | Left anterior cingulate / paracingulate gyri, BA 11(10) |
| Right middle occipital gyrus | 32,-96,2 | 13 | Right middle occipital gyrus, BA 18(13) |
| ***MDD < SZ*** | | | |
| Right lenticular nucleus, putamen | 26,12,-4 | 580 | Right striatum(295) |
|  |  |  | Right lenticular nucleus, putamen, BA 48(203) |
|  |  |  | Right insula, BA 48(38) |
|  |  |  | Right insula, BA 47(16) |
|  |  |  | Right caudate nucleus, BA 25(15) |
|  |  |  | Right lenticular nucleus, putamen(13) |
| Left lenticular nucleus, putamen | -22,6,-4 | 553 | Left lenticular nucleus, putamen, BA 48(231) |
|  |  |  | Left striatum(214) |
|  |  |  | Left lenticular nucleus, putamen(52) |
|  |  |  | Left insula, BA 48(45) |
|  |  |  | Left amygdala, BA 34(11) |
| Right median cingulate / paracingulate gyri, | 4,28,38 | 619 | Right median cingulate / paracingulate gyri, BA 32(88) |
|  |  |  | Left superior frontal gyrus, medial, BA 9(88) |
|  |  |  | Left superior frontal gyrus, medial, BA 32(77) |
|  |  |  | Left superior frontal gyrus, medial, BA 8(63) |
|  |  |  | Right superior frontal gyrus, medial, BA 9(62) |
|  |  |  | Left superior frontal gyrus, medial(57) |
|  |  |  | Right superior frontal gyrus, medial, BA 32(49) |
|  |  |  | Right superior frontal gyrus, medial, BA 8(44) |
|  |  |  | Right median cingulate / paracingulate gyri, BA 24(37) |
|  |  |  | Left median cingulate / paracingulate gyri, BA 24(26) |
|  |  |  | Left anterior cingulate / paracingulate gyri, BA 24(17) |
|  |  |  | Right anterior cingulate / paracingulate gyri, BA 24(11) |

***Abbreviations:*** ReHo = Regional Homogeneity; MDD = Major depressive disorder; SZ = Schizophrenia.

| **Table S11** Jackknife sensitivity analysis for MDD meta-analysis. | | | | | | |  | |  | |
| --- | --- | --- | --- | --- | --- | --- | --- | --- | --- | --- |
| Study | | Right superior frontal gyrus, medial | | Right middle temporal gyrus | Left hippocampus | Right inferior frontal gyrus | Left sensorimotor cortex | Bilateral occipital visual cortex | | Right striatum |
| Chen et al., 2012.a | | | Y | Y | Y | Y | Y | Y | | Y |
| Chen et al., 2012.b | | | Y | Y | Y | Y | Y | Y | | Y |
| Chen et al., 2023.a | | | Y | Y | Y | Y | Y | Y | | Y |
| Chen et al., 2023.b | | | Y | Y | Y | Y | Y | Y | | Y |
| Dai et al., 2024.a | | | Y | Y | Y | Y | Y | Y | | Y |
| Dai et al., 2024.b | | | Y | Y | Y | N | Y | Y | | Y |
| Dan et al., 2023.a | | | Y | Y | Y | Y | Y | Y | | Y |
| Dan et al., 2023.b | | | Y | Y | Y | Y | Y | Y | | Y |
| Fang et al., 2022 | | | Y | Y | Y | Y | Y | Y | | Y |
| Guo et al., 2012.a | | | N | Y | Y | Y | Y | Y | | Y |
| Guo et al., 2012.b | | | Y | Y | Y | Y | Y | Y | | Y |
| Huang et al., 2014 | | | Y | Y | Y | Y | Y | Y | | Y |
| Huang et al., 2024.a | | | Y | Y | Y | Y | Y | Y | | Y |
| Huang et al., 2024.b | | | Y | Y | Y | Y | Y | Y | | Y |
| Liang et al., 2013 | | | Y | Y | Y | Y | Y | Y | | Y |
| Liang et al., 2020 | | | Y | Y | Y | Y | Y | Y | | N |
| Li.G.Z et al., 2018 | | | Y | Y | Y | Y | Y | Y | | Y |
| Li.M et al., 2018 | | | Y | Y | Y | Y | Y | Y | | Y |
| Lin et al., 2022 | | | Y | Y | Y | Y | Y | Y | | Y |
| Liu et al., 2021.a | | | Y | Y | Y | Y | Y | Y | | Y |
| Liu et al., 2021.b | | | Y | Y | Y | Y | Y | Y | | Y |
| Liu et al., 2021 | | | Y | Y | Y | Y | Y | Y | | Y |
| Liu et al., 2017 | | | Y | Y | N | Y | Y | Y | | Y |
| Liu et al., 2010 | | | Y | Y | Y | Y | Y | Y | | Y |
| Ni et al., 2023 | | | Y | Y | Y | Y | Y | Y | | Y |
| Ni et al., 2024 | | | Y | Y | Y | Y | Y | Y | | Y |
| Peng et al., 2011 | | | Y | Y | Y | Y | Y | Y | | Y |
| Shen et al., 2017.a | | | Y | Y | Y | Y | Y | Y | | Y |
| Shen et al., 2017.b | | | Y | Y | Y | Y | Y | Y | | Y |
| Song et al., 2016 | | | Y | Y | Y | Y | Y | Y | | Y |
| Song et al., 2020 | | | Y | Y | Y | Y | Y | Y | | Y |
| Sun et al., 2022 | | | Y | N | Y | Y | Y | Y | | Y |
| Tu et al., 2022 | | | N | Y | Y | Y | Y | Y | | Y |
| Wang et al., 2013 | | | Y | Y | Y | Y | Y | Y | | Y |
| Wu et al., 2011 | | | Y | Y | Y | Y | Y | Y | | Y |
| Wu et al., 2024 | | | Y | Y | Y | Y | Y | Y | | Y |
| Yang et al., 2015 | | | Y | Y | Y | Y | Y | Y | | Y |
| Zhang.A.X et al., 2022 | | | Y | Y | Y | Y | Y | Y | | Y |
| Zhang.B et al., 2022 | | | Y | Y | Y | Y | Y | Y | | Y |
| Zhang et al., 2021 | | | Y | Y | Y | Y | Y | Y | | Y |
| Zhao et al., 2023 | | | Y | Y | Y | Y | Y | Y | | Y |
| Zhou et al., 2022.a | | | Y | Y | Y | Y | Y | Y | | Y |
| Zhou et al., 2022.b | | | Y | Y | Y | Y | Y | Y | | Y |
|  |  | | 42/43 | 42/43 | 42/43 | 42/43 | 43/43 | 43/43 | | 42/43 |

| **Table S12** Jackknife sensitivity analysis for BD meta-analysis. | | | | | | | | | |
| --- | --- | --- | --- | --- | --- | --- | --- | --- | --- |
| Study | Right insula/Right temporal pole, superior temporal gyrus/Right inferior frontal gyrus, orbital part | Left inferior frontal gyrus, orbital part | | Left precuneus | | Cerebellum, vermic lobule IV / V | | Left precentral gyrus, | |
| Adrian et al., 2021 | Y | | Y | | Y | | Y | | Y |
| Cui et al., 2016 | Y | | Y | | Y | | Y | | Y |
| Gao et al., 2014 | Y | | Y | | Y | | Y | | Y |
| Jiang et al., 2016 | Y | | Y | | Y | | Y | | Y |
| Lei et al., 2024 | Y | | Y | | Y | | Y | | Y |
| Liang et al., 2013 | Y | | Y | | Y | | Y | | Y |
| Li et al., 2020 | Y | | Y | | Y | | Y | | Y |
| Liu et al., 2012 | Y | | Y | | Y | | Y | | Y |
| Liu et al., 2020 | Y | | Y | | Y | | Y | | Y |
| Ni et al., 2024 | Y | | Y | | Y | | Y | | Y |
| Qiu et al., 2019 | Y | | Y | | Y | | Y | | Y |
| Russo et al., 2020 | Y | | Y | | Y | | N | | N |
| Shan et al., 2020 | Y | | Y | | Y | | Y | | Y |
| Wu et al., 2023 | Y | | Y | | Y | | Y | | Y |
| Xiao et al., 2013 | Y | | Y | | Y | | Y | | Y |
| Xiao et al., 2019 | Y | | Y | | Y | | Y | | Y |
| Yao et al., 2018 | Y | | N | | Y | | Y | | Y |
| Zhao et al., 2024 | Y | | Y | | Y | | Y | | Y |
|  | 18/18 | | 17/18 | | 18/18 | | 17/18 | | 17/18 |

| **Table S13** Jackknife sensitivity analysis for SZ meta-analysis. | | | | | | | | | | |
| --- | --- | --- | --- | --- | --- | --- | --- | --- | --- | --- |
| Study | Right striatum/  Right inferior frontal gyrus, triangular part, | | Left superior frontal gyrus, medial | Left angular gyrus | Right precentral gyrus | Left posterior cingulate gyrus | Right superior occipital gyrus | Left superior occipital gyrus | Right middle occipital gyrus | Left superior frontal gyrus, medial orbital |
| Chen et al., 2025 | Y | Y | | Y | Y | Y | Y | Y | Y | Y |
| Cui et al., 2016 | Y | Y | | Y | Y | Y | Y | Y | Y | Y |
| Cui et al., 2016 | Y | N | | Y | Y | Y | Y | Y | Y | Y |
| Fang et al., 2013 | Y | Y | | Y | Y | Y | Y | Y | Y | Y |
| Gao et al., 2015 | Y | Y | | Y | Y | Y | Y | Y | Y | Y |
| Gao et al., 2018.a | Y | Y | | Y | Y | Y | N | Y | Y | Y |
| Gao et al., 2018.b | Y | Y | | N | Y | Y | Y | Y | Y | Y |
| Gao et al., 2020 | Y | Y | | Y | Y | Y | Y | Y | Y | Y |
| Gou et al., 2018 | Y | Y | | Y | Y | Y | Y | Y | Y | Y |
| Huang et al., 2022 | Y | Y | | Y | Y | N | N | Y | N | N |
| Hu et al., 2016 | Y | Y | | Y | Y | Y | Y | Y | Y | Y |
| Jiang et al., 2010 | Y | Y | | Y | Y | Y | Y | Y | Y | Y |
| Jin et al., 2021 | Y | Y | | Y | Y | Y | Y | Y | Y | Y |
| Li et al., 2024 | Y | Y | | Y | Y | Y | Y | Y | Y | Y |
| Liu et al., 2016 | Y | | Y | Y | Y | Y | Y | Y | Y | Y |
| Liu et al., 2006 | Y | | Y | Y | Y | Y | Y | Y | Y | Y |
| Liu et al., 2025 | Y | | Y | Y | Y | Y | Y | Y | Y | Y |
| Shan et al., 2021 | Y | | Y | Y | Y | Y | Y | Y | Y | Y |
| Wang et al., 2017 | Y | | Y | Y | Y | Y | Y | Y | Y | Y |
| Wu et al., 2018 | Y | | Y | N | Y | Y | Y | Y | Y | Y |
| Yan et al., 2020 | Y | | Y | Y | Y | Y | Y | Y | Y | Y |
| Yu et al., 2013 | Y | | Y | Y | Y | Y | N | Y | Y | Y |
| Zhang et al., 2024 | Y | | Y | N | Y | Y | Y | Y | Y | Y |
| Zhang.R.R et al., 2018 | Y | | Y | Y | Y | Y | Y | Y | Y | Y |
| Zhang.S.Y et al., 2018 | Y | | Y | Y | Y | Y | Y | Y | Y | Y |
| Zhang et al., 2017 | Y | | Y | Y | Y | Y | Y | Y | Y | Y |
| Zhang et al., 2019 | Y | | Y | Y | Y | Y | Y | Y | Y | Y |
| Zhao et al., 2019 | Y | | Y | Y | Y | Y | Y | Y | Y | Y |
| Zhou et al., 2024 | Y | | Y | Y | Y | Y | Y | Y | Y | Y |
|  | 29/29 | | 28/29 | 26/29 | 29/29 | 28/29 | 26/29 | 29/29 | 28/29 | 28/29 |

**Table S14** Reliability analyses of meta-analytic ReHo abnormalities in MDD.

| Effect anatomy/region | MNI peak coordinates  X,Y,Z | Voxels | SDM-Z value | P value |
| --- | --- | --- | --- | --- |
| ***MDD > HC*** |  |  |  |  |
| **3T-only studies** |  |  |  |  |
| Right superior frontal gyrus, medial/Right anterior cingulate / paracingulate gyri | 12,46,8 | 622 | 1.843 | 0.000067115 |
| Right inferior frontal gyrus | 54,20,12 | 197 | 1.338 | 0.001847565 |
| Right middle temporal gyrus | 38,-64,24 | 166 | 1.521 | 0.000588357 |
| Left hippocampus | -28,-28,-14 | 132 | 1.508 | 0.000624478 |
| **≤ 8 mm smoothing kernel** |  |  |  |  |
| Right superior frontal gyrus, medial/Right anterior cingulate / paracingulate gyri | 4,56,0 | 443 | 1.648 | 0.000149667 |
| Right inferior frontal gyrus, triangular part | 54,20,12 | 289 | 1.363 | 0.001439869 |
| Right middle temporal gyrus | 44,-28,0 | 203 | 1.422 | 0.000975370 |
| Left hippocampus | -28,-28,-14 | 171 | 1.567 | 0.000330269 |
| **Kendall’s W with a 27-voxel neighborhood** | |  |  |  |
| Right superior frontal gyrus, medial/Right anterior cingulate / paracingulate gyri | 12,48,10 | 705 | 1.873 | 0.000144482 |
| Right middle temporal gyrus | 38,-64,24 | 243 | 1.462 | 0.001135349 |
| Right inferior frontal gyrus | 48,18,18 | 78 | 1.321 | 0.002286255 |
| ***MDD < HC*** |  |  |  |  |
| **3T-only studies** |  |  |  |  |
| Left sensorimotor cortex/Left precentral gyrus | -56,0,26 | 826 | -2.108 | 0.000092924 |
| Right cuneus cortex | 6,-78,24 | 131 | -1.592 | 0.001527607 |
| Left middle occipital gyrus | -38,-80,-2 | 100 | -1.654 | 0.001119912 |
| Right middle occipital gyrus | 48,-78,4 | 55 | -1.557 | 0.001924992 |
| Right striatum | 24,-4,-2 | 12 | -1.464 | 0.003034532 |
| **≤ 8 mm smoothing kernel** |  |  |  |  |
| Left sensorimotor cortex/Left precentral gyrus | -56,-4,30 | 602 | -1.943 | 0.000190973 |
| Right cuneus cortex | 6,-78,24 | 180 | -1.655 | 0.000975370 |
| Right middle occipital gyrus | 50,-76,6 | 79 | -1.587 | 0.001465678 |
| Right striatum | 24,-4,-2 | 79 | -1.585 | 0.001465678 |
| Left middle occipital gyrus | -30,-78,32 | 74 | -1.503 | 0.002281070 |
| Left lenticular nucleus, putamen | -32,-8,4 | 24 | -1.437 | 0.003271937 |
| **Kendall’s W with a 27-voxel neighborhood** | |  |  |  |
| Left sensorimotor cortex/Left postcentral gyrus | -56,-2,28 | 797 | -2.206 | 0.000263214 |
| Bilateral occipital visual cortex/Left cuneus cortex | -2,-84,18 | 614 | -2.280 | 0.000149667 |
| Right striatum/Right lenticular nucleus, putamen | 26,14,-4 | 156 | -1.921 | 0.000851512 |

**Table S15** Reliability analyses of meta-analytic ReHo abnormalities in BD.

| Effect anatomy/region | MNI peak coordinates  X,Y,Z | Voxels | SDM-Z value | P value |
| --- | --- | --- | --- | --- |
| ***BD > HC*** |  |  |  |  |
| **3T-only studies** |  |  |  |  |
| Right insula/Right temporal pole, superior temporal gyrus | 46,14,-8 | 799 | 2.130 | 0.000113547 |
| Left inferior frontal gyrus, orbital part, | -46,28,-10 | 158 | 1.757 | 0.001274705 |
| **≤ 8 mm smoothing kernel** |  |  |  |  |
| Right insula/Right temporal pole, superior temporal gyrus/Right inferior frontal gyrus, orbital part | 42,10,-12 | 694 | 2.076 | 0.000159979 |
| Left inferior frontal gyrus | -46,28,-12 | 90 | 1.732 | 0.001450181 |
| **Kendall’s W with a 27-voxel neighborhood** | |  |  |  |
| Right temporal pole, superior temporal gyrus/Right inferior frontal gyrus, orbital part | 48,12,-14 | 585 | 2.281 | 0.000041306 |
| Right insula | 42,-6,-2 | 117 | 1.612 | 0.002482355 |
| ***BD < HC*** |  |  |  |  |
| **3T-only studies** |  |  |  |  |
| Left precuneus /Right precuneus | 8,-56,32 | 2201 | -3.007 | 0.000005186 |
| Cerebellum, vermic lobule IV / V | 0,-58,-10 | 192 | -2.196 | 0.000345767 |
| Left precentral gyrus | -54,-2,28 | 109 | -1.870 | 0.002275944 |
| **≤ 8 mm smoothing kernel** |  |  |  |  |
| Left precuneus | -2,-36,40 | 2355 | -2.772 | 0.000005186 |
| Cerebellum, vermic lobule IV / V | 0,-60,-6 | 201 | -2.148 | 0.000371575 |
| Left precentral gyrus | -46,4,36 | 115 | -1.798 | 0.002074659 |
| Left median network, cingulum | -14,48,4 | 42 | -1.930 | 0.001052797 |
| **Kendall’s W with a 27-voxel neighborhood** | |  |  |  |
| Left precuneus | 4,-62,24 | 798 | -2.828 | 0.000005186 |
| Cerebellum, vermic lobule IV / V | -8,-60,-4 | 330 | -2.278 | 0.000185788 |
| Left precentral gyrus/Left inferior frontal gyrus, opercular part | -48,6,24 | 71 | -1.887 | 0.002224326 |

**Table S16** Reliability analyses of meta-analytic ReHo abnormalities in SZ.

| Effect anatomy/region | MNI peak coordinates  X,Y,Z | Voxels | SDM-Z value | P value |
| --- | --- | --- | --- | --- |
| ***SZ > HC*** |  |  |  |  |
| **3T-only studies** |  |  |  |  |
| Left striatum/Right inferior frontal gyrus | -22,14,-8 | 4263 | 2.920 | ~0 |
| Medial prefrontal cortex | 10,22,40 | 1523 | 2.613 | 0.000015497 |
| Left angular gyrus | -48,-60,38 | 190 | 2.069 | 0.000459313 |
| **≤ 8 mm smoothing kernel** |  |  |  |  |
| Left striatum/ Right inferior frontal gyrus, triangular part | -22,14,-8 | 3500 | 2.993 | ~0 |
| Medial prefrontal cortex | 10,22,40 | 2234 | 2.833 | ~0 |
| **Kendall’s W with a 27-voxel neighborhood** | |  |  |  |
| Left striatum/Right inferior frontal gyrus | -22,14,-8 | 4158 | 2.988 | ~0 |
| Medial prefrontal cortex | -2,20,48 | 2022 | 2.569 | 0.000020623 |
| Left angular gyrus | -48,-60,38 | 217 | 2.180 | 0.000258029 |
| ***SZ < HC*** |  |  |  |  |
| **3T-only studies** |  |  |  |  |
| Right precentral gyrus/Somatomotor areas | 54,0,38 | 977 | -2.248 | 0.000118673 |
| Bilateral posterior cingulate cortex/Left precuneus | 4,-40,26 | 509 | -1.844 | 0.000908315 |
| Left middle occipital gyrus | -44,-78,4 | 460 | -1.980 | 0.000495434 |
| Right middle occipital gyrus | 32,-94,2 | 274 | -2.156 | 0.000206411 |
| Right superior occipital gyrus | 28,-86,32 | 278 | -2.124 | 0.000227094 |
| Left superior occipital gyrus | -14,-102,14 | 196 | -2.012 | 0.000449002 |
| **≤ 8 mm smoothing kernel** |  |  |  |  |
| Right precentral gyrus,/Right postcentral gyrus/Somatomotor areas | 56,0,38 | 1058 | -2.291 | 0.000113547 |
| Left posterior cingulate gyrus | 4,-40,26 | 609 | -1.885 | 0.000552177 |
| Right superior occipital gyrus | 28,-86,32 | 356 | -2.165 | 0.000190973 |
| Right middle occipital gyrus | 32,-96,0 | 308 | -2.123 | 0.000221908 |
| Left superior frontal gyrus, medial orbital | -4,24,-8 | 169 | -2.084 | 0.000258029 |
| Left superior occipital gyrus | -12,-102,12 | 114 | -1.862 | 0.000603795 |
| Left middle occipital gyrus | -46,-72,6 | 11 | 1.383 | 0.004541516 |
| **Kendall’s W with a 27-voxel neighborhood** | |  |  |  |
| Right precentral gyrus,/Right postcentral gyrus/Somatomotor areas | 56,-2,42 | 1430 | -2.702 | ~0 |
| Middle occipital gyrus | -46,-72,6 | 273 | -1.376 | 0.001723707 |
| Right cuneus cortex | 10,-82,38 | 231 | -1.605 | 0.000464499 |
| Posterior cingulate cortex/Left cuneus cortex/Left precuneus | -10,-66,22 | 212 | -1.490 | 0.000996053 |

**Table S17** Subgroup analysis of ReHo abnormalities in non-medicated MDD patients.

| Effect anatomy/region | MNI peak coordinates  X,Y,Z | Voxels | SDM-Z value | P value | Breakdown  (No. of voxels) |
| --- | --- | --- | --- | --- | --- |
| ***MDD > HC*** | | | | | |
| Left median network, cingulum/Left hippocampus | -28,-26,-16 | 142 | 1.637 | 0.000330269 | Left hippocampus, BA 20(76) |
|  |  |  |  |  | Left median network, cingulum(37) |
|  |  |  |  |  | Left parahippocampal gyrus, BA 20(17) |
|  |  |  |  |  | Left hippocampus, BA 37(12) |
| Right inferior frontal gyrus | 48,18,18 | 70 | 1.400 | 0.001367629 | Right inferior frontal gyrus, triangular part, BA 48(49) |
|  |  |  |  |  | Right inferior frontal gyrus, opercular part, BA 48(21) |
| Left middle frontal gyrus | -42,26,34 | 33 | 1.345 | 0.001950800 | Left middle frontal gyrus, BA 45(18) |
|  |  |  |  |  | Left middle frontal gyrus, BA 44(15) |
| Right thalamus | 10,-24,8 | 15 | 1.281 | 0.002802312 | Right thalamus(15) |
| ***MDD < HC*** | | | | | |
| Right striatum | 24,10,-2 | 504 | -2.214 | 0.000046432 | Right lenticular nucleus, putamen, BA 48(255) |
|  |  |  |  |  | Right striatum(222) |
|  |  |  |  |  | Right lenticular nucleus, putamen(27) |
| Left cuneus cortex | -2,-84,18 | 416 | -2.049 | 0.000201285 | Left cuneus cortex, BA 18(130) |
|  |  |  |  |  | Right cuneus cortex, BA 18(95) |
|  |  |  |  |  | Left calcarine fissure / surrounding cortex, BA 17(71) |
|  |  |  |  |  | Left calcarine fissure / surrounding cortex, BA 18(69) |
|  |  |  |  |  | Right calcarine fissure / surrounding cortex, BA 17(18) |
|  |  |  |  |  | Left cuneus cortex(17) |
|  |  |  |  |  | Right calcarine fissure / surrounding cortex, BA 18(16) |
| Left lenticular nucleus, putamen | -28,-2,6 | 284 | -2.116 | 0.000113547 | Left lenticular nucleus, putamen, BA 48(117) |
|  |  |  |  |  | Left insula, BA 48(62) |
|  |  |  |  |  | Left striatum(55) |
|  |  |  |  |  | Left lenticular nucleus, putamen(50) |
| Right middle occipital gyrus | 50,-78,0 | 88 | -1.610 | 0.002115905 | Right middle occipital gyrus, BA 19(65) |
|  |  |  |  |  | Right inferior occipital gyrus, BA 19(13) |
|  |  |  |  |  | Right middle temporal gyrus, BA 19(10) |

**Table S18** Subgroup analysis of ReHo abnormalities in non-medicated SZ patients.

| Effect anatomy/region | MNI peak coordinates  X,Y,Z | Voxels | SDM-Z value | P value | Breakdown  (No. of voxels) |
| --- | --- | --- | --- | --- | --- |
| ***SZ > HC*** |  |  |  |  |  |
| Right striatum | 20,2,-10 | 559 | 2.361 | ~0 | Right striatum(366) |
|  |  |  |  |  | Right amygdala, BA 34(53) |
|  |  |  |  |  | Right lenticular nucleus, putamen, BA 48(42) |
|  |  |  |  |  | Right olfactory cortex, BA 25(26) |
|  |  |  |  |  | Right olfactory cortex, BA 11(23) |
|  |  |  |  |  | Right gyrus rectus, BA 11(22) |
|  |  |  |  |  | Right caudate nucleus, BA 25(15) |
|  |  |  |  |  | Right olfactory cortex, BA 48(12) |
| Right middle frontal gyrus/Right superior frontal gyrus, dorsolateral | 26,44,38 | 252 | 1.747 | 0.000629604 | Right middle frontal gyrus, BA 9(119) |
|  |  |  |  |  | Right middle frontal gyrus, BA 46(64) |
|  |  |  |  |  | Right superior frontal gyrus, dorsolateral, BA 9(56) |
|  |  |  |  |  | Right middle frontal gyrus(13) |
| Right inferior frontal gyrus, triangular part | 46,46,0 | 151 | 1.756 | 0.000598669 | Right middle frontal gyrus, BA 46(42) |
|  |  |  |  |  | Right inferior frontal gyrus, orbital part, BA 47(37) |
|  |  |  |  |  | Right inferior frontal gyrus, triangular part, BA 45(32) |
|  |  |  |  |  | Right middle frontal gyrus, BA 45(16) |
|  |  |  |  |  | Right middle frontal gyrus, orbital part, BA 46(14) |
|  |  |  |  |  | Right middle frontal gyrus, BA 10(10) |
| Left inferior parietal | -46,-44,48 | 121 | 1.662 | 0.000980556 | Left inferior parietal gyri, BA 40(102) |
|  |  |  |  |  | Left inferior parietal gyri, BA 2(19) |
| Left precentral gyrus | -32,-12,60 | 21 | 1.514 | 0.002095282 | Left precentral gyrus, BA 6(21) |
| Right precentral gyrus | 36,-8,64 | 10 | 1.507 | 0.002270758 | Right precentral gyrus(10) |
| ***SZ < HC*** | | | | | |
| Left cuneus cortex/Left precuneus/Left median network, cingulum | 0,-70,24 | 468 | -1.694 | 0.000335455 | Left cuneus cortex(113) |
|  |  |  |  |  | Right cuneus cortex, BA 19(97) |
|  |  |  |  |  | Left precuneus, BA 23(51) |
|  |  |  |  |  | Left median network, cingulum(49) |
|  |  |  |  |  | Left cuneus cortex, BA 18(36) |
|  |  |  |  |  | Left precuneus(23) |
|  |  |  |  |  | Right cuneus cortex(23) |
|  |  |  |  |  | Right cuneus cortex, BA 7(20) |
|  |  |  |  |  | Right precuneus, BA 23(19) |
|  |  |  |  |  | Right cuneus cortex, BA 18(19) |
|  |  |  |  |  | Left cuneus cortex, BA 23(18) |
| Left middle occipital gyrus/occipital visual regions | -32,-88,0 | 155 | -1.427 | 0.001259267 | Left middle occipital gyrus, BA 19(64) |
|  |  |  |  |  | Left middle occipital gyrus, BA 18(63) |
|  |  |  |  |  | Left inferior occipital gyrus, BA 19(28) |
| Right calcarine fissure / surrounding cortex | 14,-94,0 | 26 | -1.197 | 0.004025459 | Right calcarine fissure / surrounding cortex, BA 17(26) |
| Right inferior temporal gyrus | 42,-62,-10 | 28 | -1.196 | 0.004040897 | Right inferior temporal gyrus, BA 37(28) |

**Figure S1** Shared ReHo results across different conjunction sequences.

***Abbreviations****:* ReHo = Regional Homogeneity; MDD = Major depressive disorder; BD = Bipolar Disorder; SZ = Schizophrenia*.*

**Figure S2** Common and distinct patterns of ReHo abnormalities between MDD and BD.

***Abbreviations****:* ReHo = Regional Homogeneity*; MDD = Major depressive disorder; BD = Bipolar Disorder.*

**Figure S3** Common and distinct patterns of ReHo abnormalities between BD and SZ.

***Abbreviations*:** ReHo = Regional Homogeneity*;* BD = Bipolar Disorder; SZ = Schizophrenia.

**Figure S4** Common and distinct patterns of ReHo abnormalities between MDD and SZ.

***Abbreviations*:** ReHo = Regional Homogeneity; MDD = Major depressive disorder; SZ = Schizophrenia.

**Figure S5** Meta-regression findings in MDD.

Effect Size

| Age | Left middle occipital gyrus(cluster 1) |
| --- | --- |
| 24 | 0 |
| 24 | 0 |
| 37.37 | -0.556426 |
| 44.98 | -0.293198 |
| 30.44 | 0 |
| 30.89 | 0 |
| 30.36 | 0 |
| 29.53 | 0 |
| 28.48 | 0 |
| 26 | 0 |
| 26.37 | 0 |
| 28.21 | 0 |
| 20.97 | 0 |
| 20.69 | 0 |
| 35.27 | 0.097534 |
| 34.54 | 0 |
| 33.05 | 0 |
| 28.8 | -0.307569 |
| 36.83 | 0 |
| 30.88 | 0 |
| 32.41 | -0.480399 |
| 26.87 | 0 |
| 38.25 | 0 |
| 29.67 | 0 |
| 29.08 | 0 |
| 27.84 | 0 |
| 33.9 | 0 |
| 24.31 | 0 |
| 36.78 | -0.285203 |
| 34.25 | 0 |
| 44.71 | 0 |
| 31.6 | 0 |
| 29.1 | 0 |
| 33.54 | 0 |
| 33.74 | 0 |
| 45.24 | -0.09545 |
| 31.21 | 0 |
| 34.99 | 0 |
| 20.04 | 0 |
| 31.02 | 0 |
| 30.7 | 0 |
| 14.72 | 1.03573 |
| 14.96 | 0.921987 |

Effect Size

| Age | Right middle occipital gyrus |
| --- | --- |
| 24 | 0 |
| 24 | 0 |
| 37.37 | 0 |
| 44.98 | 0 |
| 30.44 | 0 |
| 30.89 | 0 |
| 30.36 | 0 |
| 29.53 | 0 |
| 28.48 | 0 |
| 26 | 0 |
| 26.37 | 0 |
| 28.21 | 0 |
| 20.97 | 0 |
| 20.69 | 0 |
| 35.27 | 0.240311 |
| 34.54 | 0 |
| 33.05 | 0 |
| 28.8 | 0.365912 |
| 36.83 | 0 |
| 30.88 | 0 |
| 32.41 | 0 |
| 26.87 | 0 |
| 38.25 | -0.349376 |
| 29.67 | 0 |
| 29.08 | 0 |
| 27.84 | 0 |
| 33.9 | 0 |
| 24.31 | 0 |
| 36.78 | -0.359657 |
| 34.25 | 0 |
| 44.71 | 0 |
| 31.6 | 0 |
| 29.1 | 0.634167 |
| 33.54 | -0.249219 |
| 33.74 | 0 |
| 45.24 | -0.881426 |
| 31.21 | 0 |
| 34.99 | -0.144027 |
| 20.04 | 0 |
| 31.02 | 0 |
| 30.7 | 0 |
| 14.72 | 0 |
| 14.96 | 0.954037 |

Effect Size

| Age | Right postcentral gyrus |
| --- | --- |
| 24 | 0 |
| 24 | 0 |
| 37.37 | 0 |
| 44.98 | -0.663986 |
| 30.44 | 0 |
| 30.89 | 0 |
| 30.36 | 0 |
| 29.53 | 0 |
| 28.48 | 0 |
| 26 | 0 |
| 26.37 | 0 |
| 28.21 | -0.118332 |
| 20.97 | 0 |
| 20.69 | 0 |
| 35.27 | -0.349039 |
| 34.54 | -0.48217 |
| 33.05 | 0 |
| 28.8 | 0 |
| 36.83 | 0 |
| 30.88 | 0.712752 |
| 32.41 | 0 |
| 26.87 | 0 |
| 38.25 | 0 |
| 29.67 | 0 |
| 29.08 | 0 |
| 27.84 | 0 |
| 33.9 | 0 |
| 24.31 | 0 |
| 36.78 | 0.217853 |
| 34.25 | 0 |
| 44.71 | 0 |
| 31.6 | 0 |
| 29.1 | 0 |
| 33.54 | 0 |
| 33.74 | 0 |
| 45.24 | -0.794179 |
| 31.21 | 0 |
| 34.99 | 0 |
| 20.04 | 0 |
| 31.02 | 0 |
| 30.7 | 0 |
| 14.72 | 0 |
| 14.96 | 0 |

Effect Size

| Age | Left cuneus cortex |
| --- | --- |
| 24 | 0 |
| 24 | 0 |
| 37.37 | 0 |
| 44.98 | 0 |
| 30.44 | 0 |
| 30.89 | 0 |
| 30.36 | -0.706368 |
| 29.53 | 0 |
| 28.48 | 0 |
| 26 | 0 |
| 26.37 | 0 |
| 28.21 | 0.100548 |
| 20.97 | 0 |
| 20.69 | 0 |
| 35.27 | 0 |
| 34.54 | -0.309079 |
| 33.05 | 0 |
| 28.8 | 0 |
| 36.83 | 0 |
| 30.88 | 0 |
| 32.41 | 0 |
| 26.87 | 0 |
| 38.25 | -0.976069 |
| 29.67 | 0 |
| 29.08 | 0 |
| 27.84 | 0.086604 |
| 33.9 | -0.194816 |
| 24.31 | 0 |
| 36.78 | 0 |
| 34.25 | 0 |
| 44.71 | 0 |
| 31.6 | 0 |
| 29.1 | 0 |
| 33.54 | -0.129318 |
| 33.74 | 0 |
| 45.24 | -0.810023 |
| 31.21 | 0 |
| 34.99 | 0 |
| 20.04 | 0 |
| 31.02 | 0 |
| 30.7 | 0 |
| 14.72 | 0 |
| 14.96 | 0 |

***Note:*** The left middle occipital gyrus findings associated with age (MNI coordinates: −38, −86, 4; 650 voxels) and onset age (MNI coordinates: −38, −86, 4; 1071 voxels) were derived from separate meta-regression models and should be interpreted as distinct clusters despite sharing the same anatomical label.

Effect Size

| Age | Right middle temporal gyrus |
| --- | --- |
| 24 | 0 |
| 24 | 0 |
| 37.37 | 0 |
| 44.98 | 0 |
| 30.44 | 0 |
| 30.89 | 0 |
| 30.36 | 0 |
| 29.53 | 0 |
| 28.48 | 0 |
| 26 | 0 |
| 26.37 | 0 |
| 28.21 | 0 |
| 20.97 | 0 |
| 20.69 | -0.67391 |
| 35.27 | 0 |
| 34.54 | 0 |
| 33.05 | 0 |
| 28.8 | 0 |
| 36.83 | 0 |
| 30.88 | 0 |
| 32.41 | 0 |
| 26.87 | -0.082103 |
| 38.25 | 0 |
| 29.67 | 0 |
| 29.08 | 0 |
| 27.84 | 0 |
| 33.9 | 0 |
| 24.31 | 0 |
| 36.78 | 0 |
| 34.25 | 0 |
| 44.71 | 0 |
| 31.6 | 0.240263 |
| 29.1 | 0 |
| 33.54 | 0 |
| 33.74 | 0 |
| 45.24 | 0 |
| 31.21 | 0 |
| 34.99 | 0 |
| 20.04 | -0.421032 |
| 31.02 | 0 |
| 30.7 | 0 |
| 14.72 | 0 |
| 14.96 | 0 |

Effect Size

| Illness duration | Right superior frontal gyrus, medial orbital |
| --- | --- |
| 0.28 | 0 |
| 0.49 | 0 |
| 2.66 | 0 |
| 2.42 | 0 |
| 1.13 | 0.521782 |
| 1.13 | 0.143614 |
| 0.49 | 0 |
| 0.56 | 0 |
| 1.8 | 0 |
| 2.29 | 0 |
| 0.25 | 0 |
| 3.5 | 0 |
| 3.7 | 0 |
| 2.36 | 0 |
| 1.85 | 0 |
| 0.98 | 0 |
| 5.44 | -1.45212 |
| 0.5 | 0 |
| 0.43 | 0 |
| 2 | 0 |
| 1.1 | 0 |
| 2.95 | 0 |
| 2.49 | 0 |
| 0.26 | 0 |
| 0.75 | 0 |
| 0.69 | 0 |
| 2.84 | 0 |
| 1.79 | 0 |
| 0.85 | 0.493185 |
| 0.51 | 0 |
| 5.63 | 0.069081 |
| 0.82 | 0 |
| 1.78 | 0 |
| 0.5 | 0 |
| 1.67 | 0 |
| 0.68 | 0 |

Effect Size

| onset age | Left middle occipital gyrus (cluster 2) |
| --- | --- |
| 24.07 | 0 |
| 67.53 | 0 |
| 35.75 | -0.556426 |
| 42.88 | -0.293198 |
| 31.38 | 0 |
| 29.48 | 0 |
| 27.05 | 0 |
| 25.06 | 0 |
| 28.09 | 0 |
| 17.3 | 0 |
| 16.6 | 0 |
| 33.7 | 0.097534 |
| 33.15 | 0 |
| 32.33 | 0 |
| 32.3 | 0 |
| 28.33 | 0 |
| 33.33 | -0.480399 |
| 24.17 | 0 |
| 28.03 | 0 |
| 26.1 | 0 |
| 25.1 | 0 |
| 34.1 | 0 |
| 23.23 | 0 |
| 36.06 | -0.285203 |
| 30.96 | 0 |
| 29.79 | 0 |
| 29.76 | 0 |
| 32.93 | 0 |
| 29.37 | 0 |
| 31.12 | 0 |
| 28.9 | 0 |
| 31.2 | 0 |
| 12.72 | 1.03573 |
| 14.28 | 0.921987 |

**Figure S6** Meta-regression findings in SZ.

***Note:*** The left middle occipital gyrus findings associated with age (MNI coordinates: −46, −72, 6; 249 voxels) and illness duration (MNI coordinates: −46, −72, 4; 466 voxels) were derived from separate meta-regression models and should be interpreted as distinct clusters despite sharing the same anatomical label.

Effect Size

| Age | Left middle occipital gyrus (cluster 1) |
| --- | --- |
| 37.31 | 0 |
| 25.42 | 0 |
| 34.05 | 0 |
| 32.18 | 0.632429 |
| 34.24 | 0 |
| 30.11 | 0 |
| 25.91 | 0 |
| 25.26 | 0 |
| 24.81 | 0 |
| 16.35 | 0 |
| 31.32 | 0 |
| 16.72 | 0 |
| 26.44 | 0 |
| 24.06 | 0 |
| 28 | 0 |
| 24.84 | 0 |
| 15.64 | 0.969599 |
| 25.66 | 0 |
| 25.28 | 0 |
| 30.85 | 0 |
| 27.92 | 0 |
| 45.76 | 0 |
| 23.48 | 0 |
| 28 | 0 |
| 35.67 | 0 |
| 23.29 | 0.480878 |

Effect Size

| Illness duration | Right superior parietal gyrus |
| --- | --- |
| 12.74 | -0.425823 |
| 1.13 | 0 |
| 9.2 | 0 |
| 14 | -1.0139 |
| 7.88 | 0 |
| 2.52 | 0 |
| 1.26 | 0 |
| 1.1 | 0 |
| 0.7 | 0 |
| 0.8 | 0 |
| 0.48 | 0 |
| 1.53 | 0 |
| 2.24 | 0.844728 |
| 0.45 | 0 |
| 0 | 0 |
| 7.1 | 0 |
| 5.54 | 0 |
| 1.04 | 0.953148 |
| 14.94 | 0 |
| 0.88 | 0 |
| 0.46 | 0 |

Effect Size

| Illness duration | Left middle occipital gyrus (cluster 2) |
| --- | --- |
| 12.74 | -1.18451 |
| 1.13 | 0 |
| 9.2 | 0 |
| 14 | -1.3628 |
| 7.88 | -0.890973 |
| 2.52 | 0 |
| 1.26 | 0 |
| 1.1 | 0 |
| 0.7 | 0 |
| 0.8 | 0 |
| 0.48 | 0 |
| 1.53 | 0 |
| 2.24 | 0.985785 |
| 0.45 | 0 |
| 0 | 0 |
| 7.1 | 0 |
| 5.54 | 0 |
| 1.04 | 0 |
| 14.94 | 0 |
| 0.88 | 0 |
| 0.46 | 0 |

**Figure S7** Funnel plot of the meta-analysis in MDD.

**Figure S8** Funnel plot of the meta-analysis in BD.

**Figure S9** Funnel plot of the meta-analysis in SZ.

**REFERENCES**

(1) *Radua J, Mataix-Cols D, Phillips ML et al. A new meta-analytic method for neuroimaging studies that combines reported peak coordinates and statistical parametric maps. Eur Psychiatry. 2012;27(8):605-11*
(2) *Radua J, Rubia K, Canales-Rodríguez EJ et al. Anisotropic kernels for coordinate-based meta-analyses of neuroimaging studies. Front Psychiatry. 2014;5:13*
(3) *Radua J, Mataix-Cols D. Voxel-wise meta-analysis of grey matter changes in obsessive-compulsive disorder. Br J Psychiatry. 2009;195(5):393-402*
(4) *Irwig L, Macaskill P, Berry G, Glasziou P. Bias in meta-analysis detected by a simple, graphical test. Graphical test is itself biased. BMJ. 1998;316(7129):470; author reply 470-1*
(5) *Ioannidis JP, Patsopoulos NA, Rothstein HR. Reasons or excuses for avoiding meta-analysis in forest plots. BMJ. 2008;336(7658):1413-5*
(6) *Liu X, Klugah-Brown B, Zhang R et al. Pathological fear, anxiety and negative affect exhibit distinct neurostructural signatures: evidence from psychiatric neuroimaging meta-analysis. Transl Psychiatry. 2022;12(1):405. Published 2022 Sep 23.*
(7) *Sterne JA, Sutton AJ, Ioannidis JP et al. Recommendations for examining and interpreting funnel plot asymmetry in meta-analyses of randomised controlled trials. BMJ. 2011;343:d4002*

**INCLUDED STUDIES**

*[1] Chen JD, Liu F, Xun GL, et al. Early and late onset, first-episode, treatment-naïve depression: same clinical symptoms, different regional neural activities. J Affect Disord. 2012;143(1-3):56-63. Doi:10.1016/j.jad.2012.05.025*

*[2] Chen S, Yin Y, Yue Y, et al. Integrating functional neuroimaging and serum proteins improves the diagnosis of major depressive disorder. J Affect Disord. 2023;325:421-428. Doi:10.1016/j.jad.2023.01.034*

*[3] Chen S, Yin Y, Zhang Y, et al. Abnormal spontaneous activity of regions related to mood regulation mediates the effect of childhood emotional neglect on major depressive disorder. Psychiatry Res Neuroimaging. 2023;336:111729. Doi:10.1016/j.pscychresns.2023.111729*

*[4] Dai K, Liu X, Hu J, et al. Insomnia-related brain functional correlates in first-episode drug-naïve major depressive disorder revealed by resting-state fMRI. Front Neurosci. 2024;18:1290345. Published 2024 Aug 29. Doi:10.3389/fnins.2024.1290345*

*[5] Lv D, Ou Y, Xiao D, et al. Identifying major depressive disorder with associated sleep disturbances through fMRI regional homogeneity at rest. BMC Psychiatry. 2023;23(1):809. Published 2023 Nov 7. Doi:10.1186/s12888-023-05305-7*

*[6] Fang Z, Mu Q, Wu C, et al. The impacts of anhedonia on brain functional alterations in patients with major depressive disorder: A resting-state functional magnetic resonance imaging study of regional homogeneity. J Psychiatr Res. 2022;156:84-90. Doi:10.1016/j.jpsychires.2022.10.028*

*[7] Guo WB, Liu F, Chen JD, et al. Abnormal neural activity of brain regions in treatment-resistant and treatment-sensitive major depressive disorder: a resting-state fMRI study. J Psychiatr Res. 2012;46(10):1366-1373. Doi:10.1016/j.jpsychires.2012.07.003*

*[8] Huang X, Huang P, Li D, et al. Early brain changes associated with psychotherapy in major depressive disorder revealed by resting-state fMRI: evidence for the top-down regulation theory. Int J Psychophysiol. 2014;94(3):437-444. Doi:10.1016/j.ijpsycho.2014.10.011*

*[9] Huang Y, Yan R, Zhang Y, et al. Abnormal fractional amplitude of low-frequency fluctuations and regional homogeneity in major depressive disorder with non-suicidal self-injury. Clin Neurophysiol. 2024;157:120-129. Doi:10.1016/j.clinph.2023.11.016*

*[10] Liang MJ, Zhou Q, Yang KR, et al. Identify changes of brain regional homogeneity in bipolar disorder and unipolar depression using resting-state FMRI. PLoS One. 2013;8(12):e79999. Published 2013 Dec 4. Doi:10.1371/journal.pone.0079999*

*[11] Liang S, Xue K, Wang W, et al. Altered brain function and clinical features in patients with first-episode, drug naïve major depressive disorder: A resting-state fMRI study. Psychiatry Res Neuroimaging. 2020;303:111134. Doi:10.1016/j.pscychresns.2020.111134*

*[12] Li G, Rossbach K, Zhang A, Liu P, Zhang K. Resting-state functional changes in the precuneus within first-episode drug-naïve patients with MDD. Neuropsychiatr Dis Treat. 2018;14:1991-1998. Published 2018 Aug 7. Doi:10.2147/NDT.S168060*

*[13] Li M, Xu H, Lu S. Neural Basis of Depression Related to a Dominant Right Hemisphere: A Resting-State fMRI Study. Behav Neurol. 2018;2018:5024520. Published 2018 Jun 5. Doi:10.1155/2018/5024520*

*[14] Lin Z, Xu X, Wang T, Huang Z, Wang G. Abnormal regional homogeneity and functional connectivity in major depressive disorder patients with long-term remission: An exploratory study. Psychiatry Res Neuroimaging. 2022;327:111557. Doi:10.1016/j.pscychresns.2022.111557*

*[15] Liu P, Tu H, Zhang A, et al. Brain functional alterations in MDD patients with somatic symptoms: A resting-state fMRI study. J Affect Disord. 2021;295:788-796. Doi:10.1016/j.jad.2021.08.143*

*[16] Liu S, Ma R, Luo Y, et al. Facial Expression Recognition and ReHo Analysis in Major Depressive Disorder. Front Psychol. 2021;12:688376. Published 2021 Sep 22. Doi:10.3389/fpsyg.2021.688376*

*[17] Liu Y, Zhao X, Cheng Z, et al. Regional homogeneity associated with overgeneral autobiographical memory of first-episode treatment-naïve patients with major depressive disorder in the orbitofrontal cortex: A resting-state fMRI study. J Affect Disord. 2017;209:163-168. Doi:10.1016/j.jad.2016.11.044*

*[18] Liu Z, Xu C, Xu Y, et al. Decreased regional homogeneity in insula and cerebellum: a resting-state fMRI study in patients with major depression and subjects at high risk for major depression. Psychiatry Res. 2010;182(3):211-215. Doi:10.1016/j.pscychresns.2010.03.004*

*[19] Ni S, Gao S, Ling C, et al. Altered brain regional homogeneity is associated with cognitive dysfunction in first-episode drug-naïve major depressive disorder: A resting-state fMRI study. J Affect Disord. 2023;343:102-108. Doi:10.1016/j.jad.2023.10.003*

*[20] Ni S, Peng T, Gao S, et al. Altered brain regional homogeneity, depressive symptoms, and cognitive impairments in medication-free female patients with current depressive episodes in bipolar disorder and major depressive disorder. BMC Psychiatry. 2024;24(1):892. Published 2024 Dec 6. Doi:10.1186/s12888-024-06352-4*

*[21] Peng DH, Jiang KD, Fang YR, et al. Decreased regional homogeneity in major depression as revealed by resting-state functional magnetic resonance imaging. Chin Med J (Engl). 2011;124(3):369-373.*

*[22] Shen Z, Jiang L, Yang S, et al. Identify changes of brain regional homogeneity in early and later adult onset patients with first-episode depression using resting-state fMRI. PLoS One. 2017;12(9):e0184712. Published 2017 Sep 14. Doi:10.1371/journal.pone.0184712*

*[23] Xue S, Wang X, Wang W, Liu J, Qiu J. Frequency-dependent alterations in regional homogeneity in major depression. Behav Brain Res. 2016;306:13-19. Doi:10.1016/j.bbr.2016.03.012*

*[24] Song Y, Shen X, Mu X, Mao N, Wang B. A study on BOLD fMRI of the brain basic activities of MDD and the first-degree relatives. Int J Psychiatry Clin Pract. 2020;24(3):236-244. doi:10.1080/13651501.2020.1744663*

*[25] Sun JF, Chen LM, He JK, et al. A Comparative Study of Regional Homogeneity of Resting-State fMRI Between the Early-Onset and Late-Onset Recurrent Depression in Adults. Front Psychol. 2022;13:849847. Published 2022 Apr 7. doi:10.3389/fpsyg.2022.849847*

*[26] Tu Z, Wu F, Jiang X, Kong L, Tang Y. Gender differences in major depressive disorders: A resting state fMRI study. Front Psychiatry. 2022;13:1025531. Published 2022 Nov 10. doi:10.3389/fpsyt.2022.1025531*

*[27] Wang L, Li K, Zhang Q, et al. Short-term effects of escitalopram on regional brain function in first-episode drug-naive patients with major depressive disorder assessed by resting-state functional magnetic resonance imaging. Psychol Med. 2014;44(7):1417-1426. doi:10.1017/S0033291713002031*

*[28] Wu QZ, Li DM, Kuang WH, et al. Abnormal regional spontaneous neural activity in treatment-refractory depression revealed by resting-state fMRI. Hum Brain Mapp. 2011;32(8):1290-1299. doi:10.1002/hbm.21108*

*[29] Wu X, Shen X, Li Q, Wang P. Temporal dynamic alterations of regional homogeneity in major depressive disorder: a study integrating machine learning. Neuroreport. 2024;35(15):972-979. doi:10.1097/WNR.0000000000002086*

*[30] Yang X, Ma X, Li M, et al. Anatomical and functional brain abnormalities in unmedicated major depressive disorder. Neuropsychiatr Dis Treat. 2015;11:2415-2423. Published 2015 Sep 18. doi:10.2147/NDT.S93055*

*[31] Zhang A, Wang X, Li J, et al. Resting-State fMRI in Predicting Response to Treatment With SSRIs in First-Episode, Drug-Naive Patients With Major Depressive Disorder. Front Neurosci. 2022;16:831278. Published 2022 Feb 16. doi:10.3389/fnins.2022.831278*

*[32] Zhang B, Liu S, Chen S, et al. Common and unique neural activities in subclinical depression and major depressive disorder indicate the development of brain impairments in different depressive stages. J Affect Disord. 2022;317:278-286. doi:10.1016/j.jad.2022.08.128*

*[33] Zhang Z, Chen Y, Wei W, et al. Changes in Regional Homogeneity of Medication-Free Major Depressive Disorder Patients With Different Onset Ages. Front Psychiatry. 2021;12:713614. Published 2021 Oct 1. doi:10.3389/fpsyt.2021.713614*

*[34] Zhao S, Wu J, Liu X, et al. Altered resting-state brain activity in major depressive disorder comorbid with subclinical hypothyroidism: A regional homogeneity analysis. Brain Res Bull. 2023;202:110754. doi:10.1016/j.brainresbull.2023.110754*

*[35] Zhou Y, Yu R, Ai M, et al. A Resting State Functional Magnetic Resonance Imaging Study of Unmedicated Adolescents With Non-suicidal Self-Injury Behaviors: Evidence From the Amplitude of Low-Frequency Fluctuation and Regional Homogeneity Indicator. Front Psychiatry. 2022;13:925672. Published 2022 Jun 17. doi:10.3389/fpsyt.2022.925672*

*[36] Chrobak AA, Bohaterewicz B, Sobczak AM, et al. Time-Frequency Characterization of Resting Brain in Bipolar Disorder during Euthymia-A Preliminary Study. Brain Sci. 2021;11(5):599. Published 2021 May 7. doi:10.3390/brainsci11050599*

*[37] CUI, DONG, GAO, WEIJIA, JIAO, QING, et al. Abnormal Resting-State Regional Homogeneity Relates to Cognitive Dysfunction in Manic Bipolar Disorder Adolescents: An fMRI Study[J]. Journal of Medical Imaging and Health Informatics,2016,6(7):1673-1678. DOI:10.1166/jmihi.2016.1870.*

*[38] Gao W, Jiao Q, Lu S, et al. Alterations of regional homogeneity in pediatric bipolar depression: a resting-state fMRI study. BMC Psychiatry. 2014;14:222. Published 2014 Aug 6. doi:10.1186/s12888-014-0222-y*

*[39] Jiang XW, Zhou Q, Tang YQ, et al. Regional homogeneity of resting-state brain function in patients with bipolar disorder in depressive phase: a functional magnetic resonance imaging study. Zhongguo Yi Ke Da Xue Xue Bao. 2016;45(4):305-308. doi:10.12007/j.issn.0258-4646.2016.04.005*

*[40] Zhao L, Bo Q, Zhang Z, Li F, Zhou Y, Wang C. Disrupted default mode network connectivity in bipolar disorder: a resting-state fMRI study. BMC Psychiatry. 2024;24(1):428. Published 2024 Jun 7. doi:10.1186/s12888-024-05869-y*

*[41] Liang MJ, Zhou Q, Yang KR, et al. Identify changes of brain regional homogeneity in bipolar disorder and unipolar depression using resting-state FMRI. PLoS One. 2013;8(12):e79999. Published 2013 Dec 4. doi:10.1371/journal.pone.0079999*

*[42] Li H, Cui L, Cao L, et al. Identification of bipolar disorder using a combination of multimodality magnetic resonance imaging and machine learning techniques. BMC Psychiatry. 2020;20(1):488. Published 2020 Oct 6. doi:10.1186/s12888-020-02886-5*

*[43] Liu CH, Ma X, Li F, et al. Regional homogeneity within the default mode network in bipolar depression: a resting-state functional magnetic resonance imaging study. PLoS One. 2012;7(11):e48181. doi:10.1371/journal.pone.0048181*

*[44] Liu P, Li Q, Zhang A, et al. Similar and Different Regional Homogeneity Changes Between Bipolar Disorder and Unipolar Depression: A Resting-State fMRI Study. Neuropsychiatr Dis Treat. 2020;16:1087-1093. Published 2020 Apr 28. doi:10.2147/NDT.S249489*

*[45] Ni S, Peng T, Gao S, et al. Altered brain regional homogeneity, depressive symptoms, and cognitive impairments in medication-free female patients with current depressive episodes in bipolar disorder and major depressive disorder. BMC Psychiatry. 2024;24(1):892. Published 2024 Dec 6. doi:10.1186/s12888-024-06352-4*

*[46] Qiu S, Chen F, Chen G, et al. Abnormal resting-state regional homogeneity in unmedicated bipolar II disorder. J Affect Disord. 2019;256:604-610. doi:10.1016/j.jad.2019.06.037*

*[47] Russo D, Martino M, Magioncalda P, Inglese M, Amore M, Northoff G. Opposing Changes in the Functional Architecture of Large-Scale Networks in Bipolar Mania and Depression. Schizophr Bull. 2020;46(4):971-980. doi:10.1093/schbul/sbaa004*

*[48] Shan X, Qiu Y, Pan P, et al. Disrupted Regional Homogeneity in Drug-Naive Patients With Bipolar Disorder. Front Psychiatry. 2020;11:825. Published 2020 Aug 14. doi:10.3389/fpsyt.2020.00825*

*[49] Wu J, Qi S, Yu W, Gao Y, Ma J. Regional Homogeneity of the Left Posterior Cingulate Gyrus May Be a Potential Imaging Biomarker of Manic Episodes in First-Episode, Drug-Naive Bipolar Disorder. Neuropsychiatr Dis Treat. 2023;19:2775-2785. Published 2023 Dec 12. doi:10.2147/NDT.S441021*

*[50] Xiao Q, Zhong Y, Lu D, et al. Altered regional homogeneity in pediatric bipolar disorder during manic state: a resting-state fMRI study. PLoS One. 2013;8(3):e57978. doi:10.1371/journal.pone.0057978*

*[51] Xiao Q, Cui D, Jiao Q, et al. Altered regional homogeneity in pediatric bipolar disorder during manic and euthymic state: a resting-state fMRI study. Brain Imaging Behav. 2019;13(6):1789-1798. doi:10.1007/s11682-019-00117-4*

*[52] Yao X, Yin Z, Liu F, et al. Shared and distinct regional homogeneity changes in bipolar and unipolar depression. Neurosci Lett. 2018;673:28-32. doi:10.1016/j.neulet.2018.02.033*

*[53]Zhao L, Bo Q, Zhang Z, Li F, Zhou Y, Wang C. Disrupted default mode network connectivity in bipolar disorder: a resting-state fMRI study. BMC Psychiatry. 2024;24(1):428. Published 2024 Jun 7. doi:10.1186/s12888-024-05869-y*

*[54]Chen X, Wu X, Zhang W, et al. Alterations in regional homogeneity in schizophrenia patients comorbid with metabolic syndrome treated with risperidone or clozapine. J Psychiatr Res. 2025;181:245-252. doi:10.1016/j.jpsychires.2024.11.068*

*[55]Cui LB, Liu K, Li C, et al. Putamen-related regional and network functional deficits in first-episode schizophrenia with auditory verbal hallucinations. Schizophr Res. 2016;173(1-2):13-22. doi:10.1016/j.schres.2016.02.039*

*[56]Fang L, Cao D, Xie SP, et al. A resting-state fMRI study on regional homogeneity in first-episode drug-naïve schizophrenia patients. Chin J Nerv Ment Dis. 2013;39(1):29-33. doi:10.3969/j.issn.1002-0152.2013.01.006.*

*[57] BIN, GAO, YIQUAN, WANG, WEIBO, LIU, et al. Spontaneous Activity Associated with Delusions of Schizophrenia in the Left Medial Superior Frontal Gyrus: A Resting-State fMRI Study.[J]. PloS one,2015,10e0133766. DOI:10.1371/journal.pone.0133766.*

*[58] Gao S, Lu S, Shi X, et al. Distinguishing Between Treatment-Resistant and Non-Treatment-Resistant Schizophrenia Using Regional Homogeneity. Front Psychiatry. 2018;9:282. Published 2018 Aug 6. doi:10.3389/fpsyt.2018.00282*

*[59] Gao S, Ming Y, Wang J, et al. Enhanced Prefrontal Regional Homogeneity and Its Correlations With Cognitive Dysfunction/Psychopathology in Patients With First-Diagnosed and Drug-Naive Schizophrenia. Front Psychiatry. 2020;11:580570. Published 2020 Oct 23. doi:10.3389/fpsyt.2020.580570*

*[60] Gou N, Liu Z, Palaniyappan L, et al. Effects of DISC1 Polymorphisms on Resting-State Spontaneous Neuronal Activity in the Early-Stage of Schizophrenia. Front Psychiatry. 2018;9:137. Published 2018 May 23. doi:10.3389/fpsyt.2018.00137*

*[61] Huang Y, Wang W, Hei G, et al. Altered regional homogeneity and cognitive impairments in first-episode schizophrenia: A resting-state fMRI study. Asian J Psychiatr. 2022;71:103055. doi:10.1016/j.ajp.2022.103055*

*[62] Hu ML, Zong XF, Zheng JJ, et al. Short-term Effects of Risperidone Monotherapy on Spontaneous Brain Activity in First-episode Treatment-naïve Schizophrenia Patients: A Longitudinal fMRI Study. Sci Rep. 2016;6:34287. Published 2016 Oct 4. doi:10.1038/srep34287*

*[63] Jiang SE, Zhou B, Liao YH, et al. A preliminary study of resting-state brain activity regional homogeneity in early-onset schizophrenia based on fMRI. Zhong Nan Da Xue Xue Bao Yi Xue Ban. 2010;35(9):947-951. doi:10.3969/j.issn.1672-7347.2010.09.008*

*[64] Jin K, Xu D, Shen Z, et al. Distinguishing hypochondriasis and schizophrenia using regional homogeneity: a resting-state fMRI study and support vector machine analysis. Acta Neuropsychiatr. 2021;33(4):182-190. doi:10.1017/neu.2021.9*

*[65] Li H, Zhang W, Song H, et al. Altered temporal lobe connectivity is associated with psychotic symptoms in drug-naïve adolescent patients with first-episode schizophrenia. Eur Child Adolesc Psychiatry. 2025;34(1):237-247. doi:10.1007/s00787-024-02485-9*

*[66] Liu C, Xue Z, Palaniyappan L, et al. Abnormally increased and incoherent resting-state activity is shared between patients with schizophrenia and their unaffected siblings. Schizophr Res. 2016;171(1-3):158-165. doi:10.1016/j.schres.2016.01.022*

*[67] Liu H, Liu Z, Liang M, et al. Decreased regional homogeneity in schizophrenia: a resting state functional magnetic resonance imaging study. Neuroreport. 2006;17(1):19-22. doi:10.1097/01.wnr.0000195666.22714.35*

*[68] Liu Y, Wan B, Liu Z, et al. Classifying schizophrenia using functional MRI and investigating underlying functional phenomena. Brain Res Bull. 2025;223:111279. doi:10.1016/j.brainresbull.2025.111279*

*[69] Shan X, Liao R, Ou Y, et al. Increased regional homogeneity modulated by metacognitive training predicts therapeutic efficacy in patients with schizophrenia. Eur Arch Psychiatry Clin Neurosci. 2021;271(4):783-798. doi:10.1007/s00406-020-01119-w*

*[70] Wang S, Zhang Y, Lv L, et al. Abnormal regional homogeneity as a potential imaging biomarker for adolescent-onset schizophrenia: A resting-state fMRI study and support vector machine analysis. Schizophr Res. 2018;192:179-184. doi:10.1016/j.schres.2017.05.038*

*[71] Wu F, Zhang Y, Yang Y, et al. Structural and functional brain abnormalities in drug-naive, first-episode, and chronic patients with schizophrenia: a multimodal MRI study. Neuropsychiatr Dis Treat. 2018;14:2889-2904. Published 2018 Oct 30. doi:10.2147/NDT.S174356*

*[72] Yan W, Zhang R, Zhou M, et al. Relationships between abnormal neural activities and cognitive impairments in patients with drug-naive first-episode schizophrenia. BMC Psychiatry. 2020;20(1):283. Published 2020 Jun 5. doi:10.1186/s12888-020-02692-z*

*[73] Yu R, Hsieh MH, Wang HL, et al. Frequency dependent alterations in regional homogeneity of baseline brain activity in schizophrenia. PLoS One. 2013;8(3):e57516. doi:10.1371/journal.pone.0057516*

*[74] Zhang R, Ren J, Lei X, et al. Aberrant patterns of spontaneous brain activity in schizophrenia: A resting-state fMRI study and classification analysis. Prog Neuropsychopharmacol Biol Psychiatry. 2024;134:111066. doi:10.1016/j.pnpbp.2024.111066*

*[75] Zhang RR, Yan W, Lyu LL, et al. Altered regional homogeneity and cognitive function in first-episode schizophrenia patients. J Clin Psychiatry. 2018;28(4):222-225. doi:10.3969/j.issn.1005-3220.2018.04.002*

*[76] Zhang S, Hu Q, Tang T, et al. Changes in Gray Matter Density, Regional Homogeneity, and Functional Connectivity in Methamphetamine-Associated Psychosis: A Resting-State Functional Magnetic Resonance Imaging (fMRI) Study. Med Sci Monit. 2018;24:4020-4030. Published 2018 Jun 13. doi:10.12659/MSM.905354*

*[77] Zhang XX, Li J, Liao JM, Jiang SS, Yan J, Yan H, Zhang D. Gray matter volume and regional homogeneity in patients with schizophrenia and obsessive-compulsive disorder. Zhongguo Xin Li Wei Sheng Za Zhi. 2017;31(10):768-774.*

*[78] Zhang Y, Guo G, Tian Y. Increased Temporal Dynamics of Intrinsic Brain Activity in Sensory and Perceptual Network of Schizophrenia. Front Psychiatry. 2019;10:484. Published 2019 Jul 12. doi:10.3389/fpsyt.2019.00484*

*[79] Zhao X, Yao J, Lv Y, et al. Abnormalities of regional homogeneity and its correlation with clinical symptoms in Naïve patients with first-episode schizophrenia. Brain Imaging Behav. 2019;13(2):503-513. doi:10.1007/s11682-018-9882-4*

*[80] Zhou Y, Zhu H, Hu W, et al. Abnormal regional homogeneity as a potential imaging indicator for identifying adolescent-onset schizophrenia: Insights from resting-state functional magnetic resonance imaging. Asian J Psychiatr. 2024;98:104106. doi:10.1016/j.ajp.2024.104106*
